# Entomopathogenic fungi *Metarhizium pingshaense* increases susceptibility to insecticides in highly resistant malaria mosquitoes *Anopheles coluzzii*

**DOI:** 10.12688/wellcomeopenres.21238.1

**Published:** 2024-05-22

**Authors:** Doubé Lucien Lamy, Edounou Jacques Gnambani, Issiaka Sare, Souro Abel Millogo, Fatimata Aïcha Sodre, Moussa Namountougou, Mafalda Viana, Francesco Baldini, Abdoulaye Diabaté, Etienne Bilgo

**Affiliations:** 1 Direction Régionale de l'Ouest, Institut de Recherche en Sciences de la Santé, Bobo-Dioulasso, Houet, 545, Burkina Faso; 2 Laboratoire de Recherches et d’Enseignements en Santé et Biotechnologies Animales (LARESBA), Universite NAZI BONI, Bobo-Dioulasso, Hauts-Bassins Region, 1091, Burkina Faso; 3 Centre Muraz, Institut National Santé Publique, Bobo-Dioulasso, Hoeut, Burkina Faso; 4 Laboratoire d’Entomologie Fondamentale et Appliquée (LEFA), Université Joseph Ki-Zerbo, Ouagadougou, Centre Region, Burkina Faso; 5 School of Biodiversity One Health and Veterinary Medicine, University of Glasgow, Glasgow, Scotland, G12 8QQ, UK; 6 Environmental Health, and Ecological Sciences Department, Ifakara Health Institute, Ifakara, Morogoro Region, Tanzania

**Keywords:** Metarhizium pingshaense, Deltamethrin, Anopheles coluzzii, Insecticide resistance, Integrated vector control, Burkina Faso

## Abstract

**Background:**

*Metarhizium* spp. based mosquito control products are among the most investigated and could potentially serve as promising complements to chemical insecticides. However, limited knowledge exists on the implementation of this biocontrol tool in conjunction with primary insecticide-based strategies to achieve synergy.

**Methods:**

In laboratory bioassays, we combined 10 ^7^ conidia/ml natives *Metarhizium pingshaense* strains with deltamethrin standard dose in three ways, before, after or simultaneously. These combinations were tested on laboratory insecticide resistant *Anopheles coluzzii*.

**Results:**

Therefore, we found that *Metarhizium pingshaense* and deltamethrin could be combined to achieve greater mortality against a highly insecticide resistance colony of *Anopheles coluzzii*. When mosquitoes were exposed to both simultaneously, no effect was observed, as expected for an insecticide resistant colony. However, when fungi were applied earlier than deltamethrin, mosquitoes became more sensitive to insecticide with a minimum Lethal Time to kill at least 50% of mosquito population (LT50) less than 8 days. In addition, when deltamethrin exposure was followed by *Metarhizium* infection, mosquito survival was similar to *Metarhizium* alone LT50 (LT50 ~11 days).

**Conclusions:**

These findings suggest that early mosquito infection to Metarhizium pingshaense followed by chemical insecticide exposure synergically improve mosquito control in the laboratory.

## Plan Language Summary

Mosquito control products made from a type of fungus called *Metarhizium* are being investigated as these can kill mosquitoes that transmit malaria, and could work together with traditional chemical insecticide. This is particularly important now as mosquitoes are becoming resistant to these chemicals and new solutions are needed. Our hypothesis is that *Metarhizium* fungi could revert insecticide resistance in mosquitoes, making them susceptible to chemicals. To prove this, we tested three ways of combining this fungus with a common chemical insecticide (deltamethrin): treating mosquito with the fungus before, after, or at the same time as the insecticide. Our tests were conducted in the laboratory using a mosquito species that transmit malaria *Anopheles coluzzii*, which is also very resistant to chemicals. We found that treating mosquitoes with fungus and then exposing them with the insecticide makes them more likely to be killed by the chemical (50% were killed only 8 days after treatment). Our research indicates that this *Metarhizium* fungus can work together with chemical insecticides to help fight against mosquitoes that have become resistant to our usual insecticides, offering a new way to tackle malaria spread.

## Background

From 2000 to 2015, we assisted in a stable and steady reduction in the malaria burden, including morbidity, mortality, and even the population at risk, with significant changes in its geographic distribution and an overall reduction of approximately 60% in terms of malaria mortality ^1, 2^. Interventions such as Long Lasting Insecticidal Nets (LLINs), Indoor Residual Spraying (IRS), and chemoprophylaxis intensively and jointly used since the early 2000 have led to impressive advances in malaria control ^3^. However, since 2015, this progress has stopped, and recurrence was observed in 2019 ^4^.

At least 80% of the progress in malaria control has been attributed to malaria prevention, primarily through vector control measures based on chemical insecticides ^3, 5^. The spread of insecticide resistance in malaria vectors has led to a drop in the effectiveness of these tools, resulting in a resurgence of the disease since 2015 ^6– 9^. The only insecticides qualified by World Health Organisation (WHO) for ITNS were pyrethroids which are highly lethal to susceptible mosquitoes after even transient contact. The massive deployment of ITNs especially in sub-Saharan Africa, with 19 billion ITNs supplied to the region from 2004 to 2019 led to insecticide resistance development in malaria mosquitoes ^10, 11^. Given the importance of insecticide-based interventions for malaria control, development of strategies to avert the selection of resistance or to control resistant mosquitoes is paramount. Co-treated ITNs with piperonyl butoxide (PBO) have already been rolled out. Although are useful in some population, but might be less effective in other populations that are very strongly resistant ^12^. Other approaches might include deployment of different insecticides in rotations or mosaics and development of novel insecticide classes ^13^. However, cross-resistance among existing insecticides and difficulty to develop new class of insecticide limit the practical options for simple chemical based insecticide resistance management approaches ^14, 15^. In this regard, the necessity to develop strategies to address insecticide resistance in malaria vector control still crucial.

Several vector control tools and strategies are being developed, including new insecticides, genetically modified mosquitoes, paratransgenesis, and entomopathogenic organisms such as fungi to address or manage insecticide resistance in malaria control ^5, 16, 17^. However, none of these tools seems to be sufficiently stand-alone to bring endemic areas toward malaria elimination on their own. One solution has been identified in the integrated vector control (IVC), which is a combination of different tools and strategies for controlling a single vector ^18^. For malaria, WHO advocates IVC for insecticide resistance management to target mosquitoes. In this context, any new control strategy or tool should be tested for compatibility with tools already in use. 

Entomopathogenic fungi are probably the most common natural insect pathogens ^19^. *Metarhizium* spp. possess several properties that make them suitable for biopesticide development as they do not need to be ingested, in contrast to bacteria and viruses; indeed, fungi infect insects through simple contact, similar to chemical insecticides ^20^. They also have the advantage of not being toxic to humans, other mammals and the environment ^21, 22^.

However, compared to chemical insecticides, *Metarhizium* spp. similar to most entomopathogenic fungi tend to be less effective, act slower, and are less cost-effective, limiting their application for control ^23, 24^. Specifically, *Metarhizium* requires at least 3 days to infect mosquitoes, mortalities occur within 7 days, and certain mosquitoes survive even 14 days post-infection ^21, 25^. Thus, even infected, some mosquitoes can survive long enough to transmit *Plasmodium* to humans. Despite these limitation, it is worth noting that *Metarhizium* infection can impact the mosquito host’s physiology as early as 3 days post infection during which they could potentially increase vector’s susceptibility to insecticide ^26, 27^. Indeed, laboratory studies showed that *Metarhizium* could be combined with chemical insecticides. Obtaining ether synergistic, antagonistic, neutral or additive effects on the insect ^28^. For example, in *Spodoptera frugiperda* larvae, fungi could revert insecticide resistance, however only in specific application sequence, depending of the type of fungi and insecticide ^28^. Combination of *Metarhizium anisopliae* with the fenitrothion insecticide was also shown to be effective against *Blattella germanica* ^29^. Notably, in *Anopheles gambiae*, immediate *Metarhizium* infection showed no effect on their insecticide susceptibility. However, when the infection reach its lethal time (3 days post-infection), a subsequent deltamethrin exposure caused higher mortality ^15^.

In this study, Farenhorst and colleagues evaluated the effect of fungal infection on insecticide efficacy. We think that for field orientated design, other option should assed. For example, in the scenario were using LLINs together with indoor residual fungal treatments or fungus treated resting targets it is possible that a mosquito gets exposed to the insecticide through the LLIN before being infected with the fungus on the treated surface. Another scenario consists to combine both on single substrates such as walls or bednets. 

In this work, we tested whether locally isolated *Metarhizium* strains could be used to increase insecticide tolerance in a highly resistant colony of *Anopheles coluzzii* mosquitoes in Burkina Faso. First, we isolated a new *Metarhizium pingshaense* strain (S31) and tested its virulence. Then, we combine this and two others well characterized native *Metarhizium pingshaense* strains [S26 and S10] with the pyrethroid insecticide deltamethrin. We tested three sequential combinations, and monitored mosquitoes’ survival up to fourteen days. We found *M. pingshaense* infected mosquitoes have improved sensitivity to deltamethrin.

## Methods

### Fungal strains

Three *Metarhizium* isolates, including two *Metarhizium* (Met) isolates (S10 and S26), were collected from *Anopheles gambiae* s.l *.* in an inhabited house in Soumousso (11°04'N, 4°03'W) and in a woodpile in Bana, respectively. *Metarhizium* strains S10 and S26 were confirmed to belong to the species *Metarhizium pingshaense* through amplification and Sanger sequencing of the intron-rich region of translation elongation factor 1-α ^25^. The third strain was newly isolated from a rhizosphere collected in the village of Bana (S31). This strain was first microscopically identified based on colony appearance, conidial shape, and conidial size ^30, 31^ and then confirmed by Polymerase Chain Reaction (PCR) of the Internal Transcribed Spacer (ITS), ITS1 (5’-TCCGTAGGTGAACCTGCGG-3’) and ITS4 (5’-TCCTCCGCTTATTGATATGC-3’).

***Fungal determination formulations for bioassays*.** To evaluate the fungal isolates pathogenicity on *An. coluzzii*, a suspension of each isolate was prepared at a concentration of 10 ^7^ conidia/mL. This concentration allows each mosquito to be infected with an average of 211 ± 13 spores ^32^. The concentration was determined using a microscope (LEICA BME) and an Improved Neubauer haemocytometer ^30, 33^. Briefly, this technique involved suspending *Metarhizium* conidia in 0.05% Tween 80 (Polysorbate Tween® 80), followed by homogenisation using a vortex (Vortex Genie 2) for 5 min. The solution was then filtered through a single-layer filter sheet. To determine the concentration, the previously obtained suspension was diluted 1:10 with 0.05% Tween 80 solution, homogenised, then 10 μL was introduced into an Improved Neubauer haemocytometer. Conidia were counted using a microscope at magnification 400X. An improved counting method was used to determine the stock solution concentration as described by Zhang *et al.* ^33^.

Insecticide-impregnated sheets with a discriminating concentration of 0.05% deltamethrin were obtained from Vector Control Research Unit, University Sains Malaysia, Penang, Malaysia.

### Mosquito strains

For the bioassays, we used pyrethroid-resistant laboratory *An. coluzzii* VKPER colony ^34, 35^. This is a pyrethroid-resistant strain that was initially collected from the Valley du Kou in Burkina Faso and then selected repeatedly to fix the *kdr* gene. Only females of 3 to 5 days old, none that had been blood fed were used and they were maintained in the laboratory under optimal conditions at 25 ± 2°C, 80% ± 10% relative humidity, and a photoperiod of 12:12 (L:D) at Institut de Recherche en Sciences de la Santé (IRSS).

### Bioassays

***Initial fungal bioassay to determine the most virulent strain effective against mosquitoes*.** Approximately 100 mosquitoes (4 replicates of 25 mosquitoes each) were infected with each of the 3 fungal isolates of *Metarhizium pingshaense* (S10, S26, and S31) or a conidia-free control Tween 80 solution. For both treated and control mosquitoes, each batch of mosquitoes was placed in cold sleep at -20°C for 15 seconds and then sprayed with the fungal solution or 0.05% Tween 80 solution for the controls before their reanimation. After spraying, the mosquitoes were allowed to reanimate. Then, mosquitoes were fed with 5% glucose solution and kept at 25°C ± 2°C and 80% ± 10% relative humidity. Mosquito mortality was monitored for 14 days after infection. As the extrinsic incubation period of *Plasmodium falciparum* is usually 10–12 days, we assessed the mortality for up to 14 days to determine the transmission potential of mosquitoes after *Metarhizium* infection. Dead mosquitoes were collected twice a day. Cadavers were immediately removed and individually washed for 20 seconds with 1% sodium hypochlorite, followed by sterile distilled water for 40 seconds, placed on agar, and incubated in the dark at 27°C. Fungal growth was assessed three days later and used to confirm the success of the fungal infection ^36^.

***Natives *Metarhizium pingshaense* and deltamethrin compatibility test*.** Three approaches were established to assess the most virulent *M. pingshaense* and deltamethrin combination against insecticide-resistant *An. coluzzii* and compared to *M. pingshaense* infection only. Simultaneous approach: a fungal suspension (10 ^7^ conidia/ml) was sprayed onto a 0.05% deltamethrin sheet and allowed to dry for 30 minutes; mosquitoes were then exposed to the sheet using a WHO insecticide susceptibility test tubes for one hour ^37^. Fungi after insecticide: mosquitoes were exposed to 0.05% deltamethrin for one hour using test WHO tubes, and after 24 hours alive mosquitoes were infected with *M. pingshaense*. Fungi before insecticide: mosquitoes were sprayed with fungi (10 ^7^ conidia/ml) and after 3 days alive mosquitoes were exposed to 0.05% deltamethrin using test tubes. Fungi only: a fungal suspension (10 ^7^ conidia/ml) was sprayed to mosquitoes as described before. In all approaches mosquito mortality was measured twice daily until 14 days after the second treatment and consisted of four replicates of 25 mosquitoes each.

### Data analysis

Data were entered into Microsoft Windows Excel 2016, checked for accuracy, and imported into RStudio version 2.11.1 for data manipulation, visualisation, and statistical analysis. Mortality of mosquitoes 14 days after exposure to fungi was analysed using generalized linear mixed models using binomial distribution, fungal strain (control, S10, S26, and S31) as fixed effect and replicates as random effect. Survival over several days after treatment was analysed using cox proportion hazard models using treatment (control, deltamethrin, S10, S26, and S31) and sequence (alone, fungi before deltamethrin, fungi after deltamethrin, fungi with deltamethrin) as fixed effects, and replicates as random effect. Model simplification was performed using Likelihood Ratio Tests (LRT) with p value < 0.05 as cutoff.

## Results

### Native strains of *Metarhizium pingshaense* virulence against insecticide-resistance mosquito line *Anopheles coluzzii*

**Figure 1.**
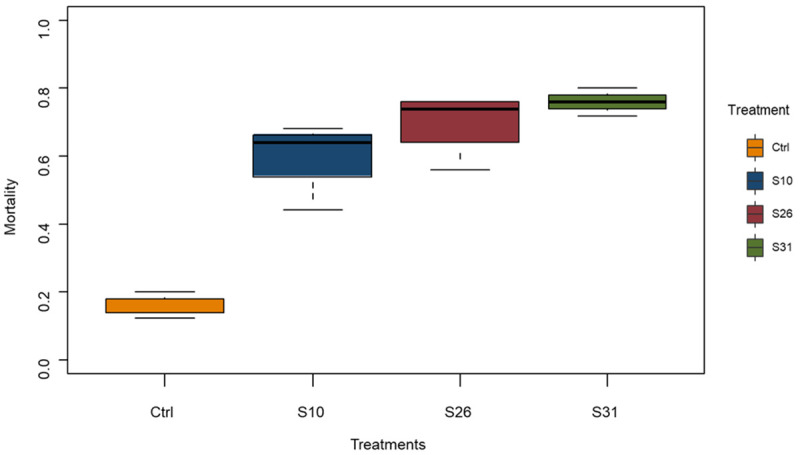
Mortality repartition of insecticide-resistant *An. coluzzii* VKPER infected with native *Metarhizium* strains, 14 days after treatment. Three *Metarhizium* strains were used: two reference strains (S10 and S26), and a newly isolated strain (S31). Virulence against the insecticide-resistant malaria vector *Anopheles coluzzii* VKPER.

Adult female *Anopheles coluzzii* aged 3–5 days were exposed to a newly isolated *Metarhizium pingshaense* strain S31 and two previously isolated strains (S10 and S26) at concentration of 1 × 10 ^7^ conidia/ml and 14-day post-infection survival compared to a control solution. All fungal treatments significantly increase mortality of mosquitoes compared to control ( Figure 1). Mosquitoes had 7.9, 12.2 and 16.6 times the odds of dying with S10, S26 and S31 respectively, compared to control (χ ^2^ = 102.525, df = 3, p < 0.001), suggesting that the newly isolated strain S31 is the most virulent of all.

### Survival of mosquitoes following the three types of combination sequences [ *M. pingshaense* and Deltamethrin 0.05%]

**Figure 2.**
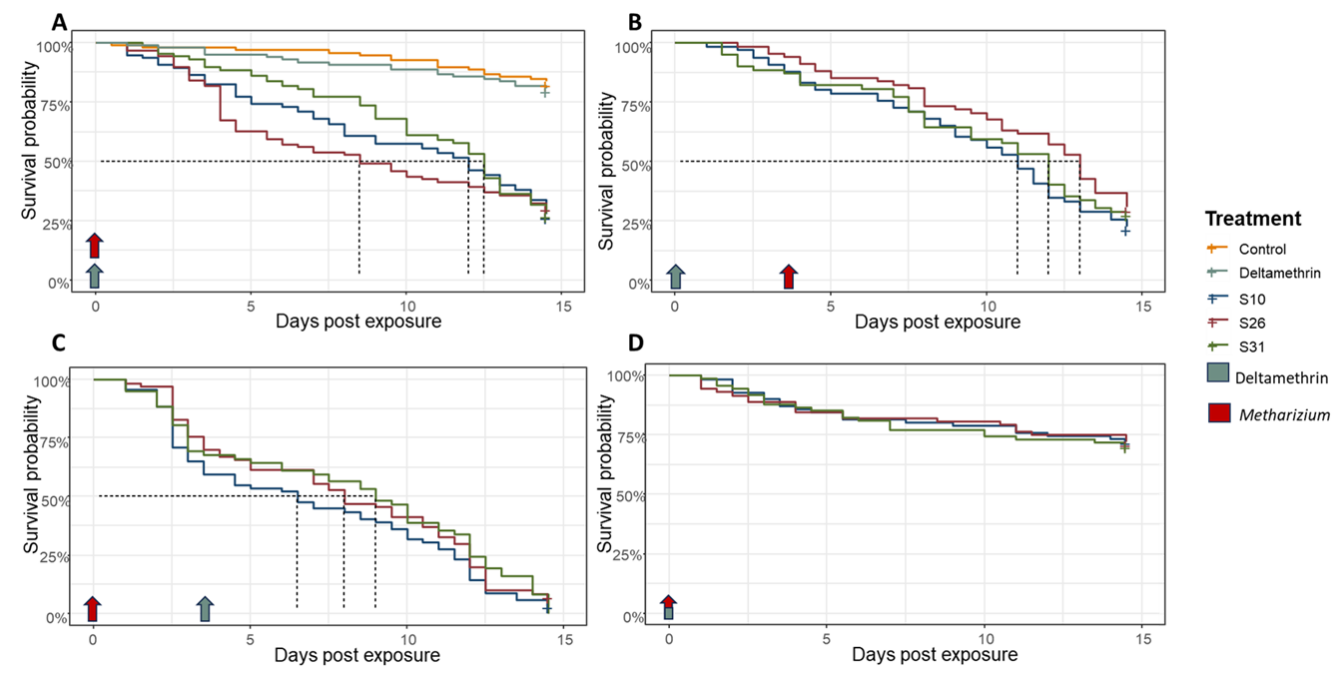
Survival curves representing mortality of mosquitoes treated with native *Metarhizium pingshaense* strains at 1×10 ^7^ conidia/ml and deltamethrin separately or in combination. Survival was measured after the first treatment fungi or deltamethrin was applied [ **A**] 3 to 5 days old mosquitoes survival to fungi strains or deltamethrin treatment; [ **B**] 3 to 5 days old mosquitoes survival after deltamethrin exposure that were infected three days later with *Metarhizium*; [ **C**] 3 to 5 days old mosquitoes infected with *Metarhizium* that were exposed to deltamethrin three days later; [ **D**] 3 to 5 days old mosquitoes exposed to a co-formulation of fungi and deltamethrin. Blue and red arrows indicate time of exposure to deltamethrin or *Metarhizium,* respectively.

We then tested if these locally isolated fungal strains could increase susceptibility to insecticides in a highly resistant strain of *Anopheles coluzzii*. We used three approaches: 1) simultaneous, where mosquitoes were exposed to a standard dose of deltamethrin and 10 ^7^ conidia/ml dose of fungi at the same time; 2) fungi after insecticide, where the same fungal dose was applied to mosquitoes 24 hours after exposure to standard deltamethrin; 3) fungi before insecticide, where mosquitoes were exposed to standard deltamethrin three days after being infected with the same fungal dose. Finally, mosquitoes were exposed to fungi only and deltamethrin as well. Cox proportional hazard analysis showed that both treatment (fungi or insecticide, χ ^2^=145.05, df=4, p < 2.2e-16) and the sequence of application (χ ^2^ = 217.14, df = 3, p < 2.2e-16) significantly affected the mortality risk compared to the control ( Table 1). Specifically, when *Metarhizium* was applied on its own, it increased the risk of death from 6.7 to 7.5 fold depending on the strain; in contrast, deltamethrin effect was similar to control (Hazard ratio = 1.2), as expected in a highly insecticide resistant strain ( Figure 2A). When fungi were applied three days after insecticide treatment, survival analysis showed a similar pattern to the treatment of fungi applied alone ( Figure 2B), as expected as this resistant colony can well tolerate deltamethrin exposure. However, when fungi were applied three days before the insecticide, we observed a significant reduction in survival, ranging from 13.7 to 15.4 fold increase in mortality compared to control depending on the strain; LT50 ranged from 6.8 to 8 days compared to 10 to 11.8 when only fungi were used ( Figure 2C). This result suggests that fungi increased the susceptibility to deltamethrin in highly insecticide resistant mosquitoes and combining *Metarhizium* with chemicals can achieve greater mortality. Finally, when fungi and insecticide were applied simultaneously as a co-formulation, the mortality was even lower than that of fungi alone and similar to deltamethrin exposure alone ( Figure 2D), suggesting that the insecticide can have a deleterious effect on fungi and ultimately their virulence. All relative statistics to the survival analysis are summarised in Table 1.

**Table 1.** Statistics of Cox proportional hazard analysis of insecticide resistant *An. coluzzii* survival treated with insecticide or fungi only, or different sequence combinations. Replicate was included as a random effect.

Response	Fixed effect	^χ ^2^^	df	p value	Hazard Ratio	LT50
Survival	Treatment	145.05	4	< 2.2e-16	Control= 1 Deltamethrin only = 1.21 S10 only = 7.53 S26 only =6.68 S31 only =6.77 S10 after Deltam. = 7.45 S26 after Deltam. = 6.62 S31 after Deltam. = 6.70 S10 before Deltam. = 15.43 S26 before Deltam. = 13.70 S31 before Deltam. = 13.88 S10 with Deltam. = 2.13 S26 with Deltam. = 1.89 S31 with Deltam. = 1.92	Control= 28.38 Deltamethrin only = 28.79 S10 only = 11.04 S26 only = 10.08 S31 only = 11.77 S10 after Deltam. = 10.62 S26 after Deltam. = 12.38 S31 after Deltam. = 11.18 S10 before Deltam. = 6.83 S26 before Deltam. = 7.94 S31 before Deltam. = 7.98 S10 with Deltam. = 26.11 S26 with Deltam. = 25.17 S31 with Deltam. = 25.61
	Sequence	217.14	3	< 2.2e-16		

## Discussion

In this study we tested if locally isolated strains of *Metarhizium pingshaense* entomopathogenic fungi combined with the pyrethroid insecticide deltamethrin can overcome insecticide resistance in highly resistant malaria mosquito colony of *Anopheles coluzzii*. We found that the order of exposure of fungi and insecticide to mosquitoes is critical: infecting mosquitoes with *Metarhizium* three days before deltamethrin exposure can increase mortality up to ~13 fold higher than deltamethrin alone; LT50 of exposure to deltamethrin decreased from 28.79 days when applied alone to 6.83 days when used after S10 strain infection. In contrast, when fungi were applied together or after the insecticide, no major effect was observed.

Several physiological mechanisms may explain the synergism observed when mosquitoes were first infected with fungi and then exposed to insecticides. Similar results were observed by Farenhorst and colleagues, when two pathogenic fungi, *Beauveria bassiana* and *Metarhizium anisopliae* were applied before permethrin in a field population of insecticide-resistant *Anopheles gambiae* mosquitoes ^15^, where fungal metabolites may have interfered with enzymatic insecticide resistance mechanisms ^26^. Another mechanism might rely on disruption of cuticular integrity by the fungus. Indeed, highly resistant mosquitoes often rely on cuticular resistance, based on a thicker cuticle which enhances the physical and chemical barrier against insecticides ^38^. *Metarhizium* infection may disrupt the thicker cuticle of resistant mosquitoes, increasing its permeability to insecticides. The fungus produces enzymes like proteases, chitinases, and lipases, which break down proteins, chitin, and lipids in the mosquito cuticle. This joint enzymatic action might have weakened the cuticle, making mosquitoes more susceptible to insecticides and leading to swift mortality ^21^. Finally, another mechanism could rely on *Metarhizium* exploiting the mosquito host energy reserves, by increasing trehalase production for fungal growth, thus impairing mosquito metabolic resistance ^21^. Combined, cuticular, energetic, and metabolic vulnerabilities could contribute to synergistic effects with deltamethrin.

The survival of mosquitoes infected with *Metarhizium pingshaense* three days after deltamethrin exposure was similar to the survival of *Metarhizium pingshaense* applied alone. Although most of the insecticide effects of deltamethrin on mosquitoes are immediate and rapid within 24-hours post exposure, some authors have shown that exposure to the insecticide can affect mosquito survival up to 14 days after exposure ^39^. However, this delayed effect of deltamethrin does not appear to be sufficiently important to have caused a statistically significant additive effect to that of *Metarhizium* alone. In contrast, an antagonistic effect was observed between *Metarhizium* and deltamethrin were combined simultaneously. Pelizza *et al.* ^40^, have hypothesized that the insecticide can reduce the viability and vegetative growth of fungal spores; thus in our study, deltamethrin could have reduced the ability of *Metarhizium pingshaense* conidia to infect mosquitoes. In the future, a combination of sublethal deltamethrin doses could tested to reduce its deleterious effect on *Metarhizium pingshaense*.

The virulence results confirmed the pathogenicity of the native isolates of *Metarhizium spp.* from Burkina Faso against the major malaria vector, *An. coluzzii* as reported by Bilgo *et al.* ^25^ including a newly isolate S31. The similarity between the mortalities of the three tested isolates could be due to their geographical proximity or the organisms from which they were isolated. Indeed, isolates S26 and S31 were isolated from mosquitoes of the genus *Anopheles* and plant roots, respectively, in the village of Bana. Like S26, isolate S10 also came from an *Anopheles* mosquito collected from an uninhabited house in Soumousso village ^25^. However, isolates S10 and S31 were distant both geographically and in terms of their origin. At the intermediate concentration of 10 ^7^ conidia/ml, no isolate reached the 80% mortality threshold set by the World Health Organization Pesticide Evaluation System (WHOPES) for effective insect control with insecticides ^41^. Bilgo *et al*. reported a mortality rate > 80% in 14 days at the same concentration ^25^. However, a study conducted in Benin by Howard *et al*. reported similar results ^42^.

This finding could lead to a new IPM strategy for malaria vector control using *Metarhizium pingshaense* as an IRS product in conjunction with the use of LLINs. The use of *Metarhizium-impregnated* fabrics placed in strategic mosquito resting places could also be considered while maintaining the current vector control strategies. Given the efficacy of the combination fungi followed by insecticide in the lab, it would be interesting to evaluate in a scenario closer to field delivery formats in semi field experiment in malaria sphere.

## Conclusion

*Metarhizium pingshaense* could be used as an alternative to replace chemical insecticides; however, their combination may offer strong synergy by improving *Metarhizium* virulence against insecticide-resistant mosquitoes. *Metarhizium* and deltamethrin are suitable for an integrated vector control approach strongly recommended by WHO for insecticide resistance management in the fight against malaria. However, it is necessary to better understand the synergy observe in this experiment by evaluating insecticide resistance related genes expression during *Metarhizium* infection. It is appropriate to evaluate these combinations in a semi-field condition. This study provides a basis for the successful integrated use of entomopathogenic fungi and chemical insecticides.

## Ethics and consent

Ethical approval and consent were not required.

## Data Availability

Enlighten Research Data: Entomopathogenic fungi Metarhizium pingshaense increases susceptibility to insecticides in highly resistant malaria mosquitoes Anopheles coluzzii. https://doi.org/10.5525/gla.researchdata.1636 ^43^. This project contains the following underlying data: ltdata.csv [Timeline mosquito mortality dataset] Mortality.csv [Fungi virulence against *Anopheles coluzzii*] Survival_analysis.csv [Mosquito survival dataset] LT50.R [R code for mosquito LT50 analysis] Mortality.R [Fungi virulence analysis R code] Survival_analysis.R [Mosquitoes survival analysis R code] Data are available under the terms of the Creative Commons Attribution 4.0 International license (CC-BY 4.0).
