# Entomopathogenic fungi *Metarhizium pingshaense* increases susceptibility to insecticides in highly resistant malaria mosquitoes *Anopheles coluzzii*

**DOI:** 10.12688/wellcomeopenres.21238.3

**Published:** 2025-05-22

**Authors:** Doubé Lucien Lamy, Edounou Jacques Gnambani, Issiaka Sare, Souro Abel Millogo, Fatimata Aïcha Sodre, Moussa Namountougou, Mafalda Viana, Francesco Baldini, Abdoulaye Diabaté, Etienne Bilgo

**Affiliations:** 1 Direction Régionale de l'Ouest, Institut de Recherche en Sciences de la Santé, Bobo-Dioulasso, Houet, 545, Burkina Faso; 2 Laboratoire de Recherches et d’Enseignements en Santé et Biotechnologies Animales (LARESBA), Universite NAZI BONI, Bobo-Dioulasso, Hauts-Bassins Region, 1091, Burkina Faso; 3 Centre Muraz, Institut National Santé Publique, Bobo-Dioulasso, Hoeut, Burkina Faso; 4 Laboratoire d’Entomologie Fondamentale et Appliquée (LEFA), Université Joseph Ki-Zerbo, Ouagadougou, Centre Region, Burkina Faso; 5 School of Biodiversity One Health and Veterinary Medicine, University of Glasgow, Glasgow, Scotland, G12 8QQ, UK; 6 Environmental Health, and Ecological Sciences Department, Ifakara Health Institute, Ifakara, Morogoro Region, Tanzania

**Keywords:** Metarhizium pingshaense, Deltamethrin, Anopheles coluzzii, Insecticide resistance, Integrated vector control, Burkina Faso

## Abstract

**Background:**

*Metarhizium* spp. based mosquito control products are among the most investigated and could potentially serve as promising complements to chemical insecticides. However, limited knowledge exists on the implementation of this biocontrol tool in conjunction with primary insecticide-based strategies to achieve synergy.

**Methods:**

In laboratory bioassays, we combined 10 ^7^ conidia/ml native *Metarhizium pingshaense* strains with deltamethrin standard dose in two ways : before and after insecticide exposure. For comparison, some mosquitoes were exposed to deltamethrin or fungi alone. These combinations were tested on laboratory insecticide resistant *Anopheles coluzzii*.

**Results:**

We found that *Metarhizium pingshaense* and deltamethrin could be combined to achieve greater mortality against a highly insecticide resistance colony of *Anopheles coluzzii*. Specifically, when fungi were applied earlier than deltamethrin, mosquitoes became more sensitive to insecticide with a minimum Lethal Time to kill at least 50% of mosquito population (LT50) less than 8 days. In addition, when deltamethrin exposure was followed by *Metarhizium* infection, mosquito survival was similar to *Metarhizium* alone LT50 (LT50 ~11 days).

**Conclusion:**

These findings suggest that early mosquito infection to *Metarhizium pingshaense* followed by chemical insecticide exposure synergically increased mosquito susceptibility to the insecticide in the laboratory.

## Plain Language Summary

Mosquito control products that use fungal spores as active ingredients known as *Metarhizium* are currently under investigation. These have demonstrated the potential to eliminate mosquitoes that are vectors of malaria. Furthermore, these products could potentially be utilised in conjunction with conventional chemical insecticides.

This is particularly important now as mosquitoes are becoming not affected by chemicals and new solutions are needed. Our hypothesis is that these fungi could increase mosquitoes vulnerability to chemical insecticide. To prove this, we tested three ways of combining this fungus with a common chemical insecticide (deltamethrin): treating mosquito with the fungus before, after, or at the same time as the insecticide. Our tests were conducted in the laboratory using a mosquito specie that transmit malaria, which is also less vulnerable to chemicals. We found that treating mosquitoes with fungus and then exposing them with the insecticide makes them more likely to be killed by the chemical. Our research indicates that this fungus can work together with chemical insecticides to help fight against mosquitoes that have become resistant to our usual insecticides, offering a new way to tackle malaria spread.

## Background

Malaria remains one of the most significant public health challenges for Africa despite substantial national and international efforts ( WHO, 2023). Malaria control is based on both preventing transmission and promptly and effectively treating infection. Transmission potential through vector control is the primary tool for malaria control ( Lengeler, 2004). From 2000 to 2015, there was a notable 60% reduction in malaria mortality, along with declines in morbidity and the population at risk, and significant geographic redistribution ( Varo *et al.*, 2020; WHO, 2016a). Since the early 2000s, intervention such as the development and distribution of insecticide-treated nets, improved diagnostic tools, the rollout of artemisinin-based combination therapies, and increased funding for malaria programs have led to impressive advances in malaria control ( Bhatt *et al.*, 2015; Global Partnership to Roll Back Malaria, 2011; The global fund, 2024; WHO, 2019). However, since 2015, this progress has stagnated, and recurrence was observed in 2020 ( WHO, 2022).

At least 80% of the progress in malaria control has been attributed to malaria prevention, primarily through vector control measures based on chemical insecticides ( Bhatt *et al.*, 2015; Hemingway *et al.*, 2016). The resurgence of malaria since 2015 can be attributed to a combination of factors, including the spread of insecticide resistance in malaria vectors, which has diminished the effectiveness of control measures. Additionally, environmental changes such as increased rainfall and urbanization have created favorable conditions for malaria transmission. Socioeconomic factors, including poverty and limited access to healthcare, further exacerbate the situation by hindering effective prevention and treatment. These interconnected issues highlight the need for a comprehensive approach to malaria control that addresses both vector resistance and the broader determinants of health ( Alout *et al.*, 2016; Hay *et al.*, 2005; Ranson & Lissenden, 2016; WHO, 2019; Yadav *et al.*, 2014). The only insecticides qualified by World Health Organisation (WHO) for ITNS were pyrethroids which are highly lethal to susceptible mosquitoes after even transient contact. The massive deployment of ITNs especially in sub-Saharan Africa, approximately 19 billion ITNs supplied to the region from 2004 to 2019 led to insecticide resistance development in malaria mosquitoes ( Lindsay *et al.*, 2021; WHO, 2020). Given the importance of insecticide-based interventions for malaria control, development of strategies to reverse insecticide resistance or to control resistant mosquitoes is paramount. ITNs co-treated with Piperonyl Butoxide (PBO) synergist have already been rolled out to act as synergist for chemical insecticide. Although PBO nets are still effective in some mosquito populations, they show significant reduction in efficacy in other populations that are highly resistant ( Killeen, 2020; Ranson & Lissenden, 2016). Other approaches might include deployment of different insecticides in rotations or mosaics and development of novel insecticide classes ( WHO, 2012b). However, cross-resistance among existing insecticides and the difficulties and long time frames often required to develop new insecticides limits the practical options for chemical based insecticide resistance management approaches ( Mnzava *et al.*, 2015; Nauen, 2007; WHO, 2018). In this regard, the necessity to develop strategies to address insecticide resistance in malaria vector control is still crucial.

Several vector control tools and strategies are being developed, including new insecticides, genetically modified mosquitoes, paratransgenesis, and entomopathogenic organisms such as fungi to address or manage insecticide resistance in malaria control ( Hemingway *et al.*, 2016; Li, 2004; Lynch *et al.*, 2012). However, none of these tools seems to be suitably efficient to bring endemic areas toward malaria elimination on their own. One proposed solution is Integrated Vector Management (IVM), which combines various malaria control strategies and tools to manage malaria transmission effectively ( Beier *et al.*, 2008; Thomas, 2018; WHO, 2012a). For malaria, WHO advocates IVC for insecticide resistance management to target mosquitoes. In this context, any new control strategy or tool should be tested for compatibility with tools already in use. Entomopathogenic fungi are among the most common natural insect pathogens, playing a significant role in the regulation of insect populations. Studies have shown that these fungi can effectively infect and kill various insect species ( Singh *et al.*, 2017; Wang *et al.*, 2016). Other natural insect pathogens include bacteria (e.g., *Bacillus thuringiensis*), viruses (e.g., nucleopolyhedroviruses), and nematodes (e.g., *Steinernema* spp.), which can also contribute to biological control of insect pests. *Metarhizium* spp. possess several properties that make them suitable for biopesticide development as they do not need to be ingested, in contrast to bacteria and viruses. Indeed fungi infect insects through simple contact, similar to chemical insecticides ( St. Leger *et al.*, 2011). These filamentous fungi produce asexual spores called conidia, which attach to the insect cuticle, germinate, and penetrate the insect body. Once inside, *Metarhizium* proliferates by utilizing the host's tissues for nutrients and can produce toxins to evade the host's immune response. This life cycle culminates in the host's death, allowing the fungus to produce new conidia, thus continuing the cycle ( Hanel, 1982). Entomopathogenic fungi are commonly found in various natural environments. While some are parasites of insects, many also colonize the rhizosphere and act as beneficial root endophytes. They have the particularity of being able to switch from one mode of life to another (St. Leger & Wang, 2020). They typically infect mosquitoes through horizontal transmission, primarily via direct contact with spores in the environment. Once infected, the fungi can proliferate within the host, leading to significant mortality rates. Studies have shown that the rate of spread in mosquito populations can be rapid, with infection rates increasing significantly in favorable environmental conditions. For example, under optimal humidity and temperature, infected individuals can serve as sources of spores, further disseminating the fungi within the population ( Reyes-Villanueva *et al.*, 2011; Scholte *et al.*, 2004). Entomopathogenic fungi have the advantage of not being toxic to humans, other mammals and the environment. Human *Metarhizium* infections are infrequent; however, a limited number of keratitis, sinusitis and cutaneous infections have been associated with Metarhizium ( Nourrisson *et al.*, 2017).

However, compared to chemical insecticides, *Metarhizium* spp. similar to most entomopathogenic fungi tend to be less effective, act slower, and are less cost-effective, limiting their application for control ( Fang *et al.*, 2012; Zhao *et al.*, 2016). Specifically, *Metarhizium* requires at least 3 days to infect mosquitoes, mortalities occur within 7 days, and certain mosquitoes survive even 14 days post-infection ( Aw & Hue, 2017; Bilgo *et al.*, 2018). Thus, even infected, some mosquitoes can survive long enough to transmit *Plasmodium* to humans, given that the extrinsic incubation period of *P. falciparum* typically ranges from 12 to 14 days (Jefferson *et al.*, 1992; Ohm *et al.*, 2018). Despite these limitations, it is worth noting that *Metarhizium* infection can impact the mosquito host’s physiology as early as 3 days post infection during which they could potentially increase vector’s susceptibility to insecticide ( Farenhorst *et al.*, 2009; Scholte *et al.*, 2006). Indeed, laboratory studies showed that *Metarhizium* could be combined with chemical insecticides. The effect of fugus could ether be synergistic, antagonistic, neutral or additive effects on the insect ( Rivero-Borja *et al.*, 2018). Laboratory studies have demonstrated the synergistic effects of combining *Metarhizium* spp. with chemical insecticides for enhanced mosquito control. Farenhorst and colleagues reported that the combination of *Metarhizium anisopliae* with pyrethroids significantly increased mortality rates in *Anopheles* mosquitoes, achieving up to a 70% improvement over individual treatments ( Farenhorst *et al.*, 2010). Combining *Metarhizium anisopliae* with fenitrothion increased the insecticide lethality by 30% up to 92% in four days depending on the fungal doses in a *Blattella germanica* ( Khaksar *et al.*, 2022). Notably, in *Anopheles gambiae*, immediate *Metarhizium* infection showed no effect on their insecticide susceptibility. However, when the infection reached its lethal time (3 days post-infection), a subsequent deltamethrin exposure caused higher mortality ( Farenhorst *et al.*, 2010).

In that study, Farenhorst and colleagues evaluated the effect of fungal infection on insecticide efficacy. It is proposed that for field-oriented design, alternative options should be assessed. For instance, in scenarios where LLINs are used in conjunction with indoor residual fungal treatments or fungus-treated resting targets, it is conceivable that a mosquito may be exposed to the insecticide through the LLIN prior to being infected with the fungus on the treated surface. An additional scenario involves combining both elements on single substrates such as walls or bednets.

We tested whether native *Metarhizium* strains could be used to decrease insecticide tolerance in a highly resistant colony of *Anopheles coluzzii* mosquitoes in Burkina Faso. First, we isolated a new *Metarhizium pingshaense* strain (S31) and tested its virulence against insecticide resistant *An. coluzzii* VKPER. Then, we combined this and two others well characterized native *Metarhizium pingshaense* strains (S26 and S10) with the pyrethroid insecticide deltamethrin. We tested two sequential combinations, and monitored mosquitoes’ survival up to fourteen days. We found that *M. pingshaense* infected mosquitoes have improved sensitivity to deltamethrin depending on the order and timing of application of each product.

## Methods

### Mosquito strains

For the bioassays, we used pyrethroid-resistant laboratory *An. coluzzii* VKPER colony ( Namountougou *et al.*, 2012; Platt *et al.*, 2015). This is a pyrethroid-resistant colony that was initially collected from the Valley du Kou in Burkina Faso and then selected repeatedly to maintain the *kdr west* gene. Mosquitoes were maintained in the insectary at Institut de Recherche en Science de la Santé (IRSS) under optimal conditions at 25 ± 2 °C, 80% ± 10% relative humidity, and a photoperiod of 12:12 (L:D). Only females of 3 to 5 days old, never blood fed were used for bioassays.

### Fungal strains

Three *Metarhizium* strains, including two *Metarhizium* (Met) strains (S10 and S26), were isolated from *Anopheles gambiae* s.l *.* in an inhabited house in Soumousso (11°04'N, 4°03'W) and in a woodpile in Bana (11°9'41"N, 4°10'30"W), respectively. *Metarhizium* strains S10 and S26 were confirmed to belong to the species *Metarhizium pingshaense* through amplification and Sanger sequencing of the intron-rich region of translation elongation factor 1-α ( Bilgo *et al.*, 2018). The third strain was newly obtained from a rhizosphere collected in the village of Bana (S31). We collected mosquitoes from houses, and woodpiles to test for fungal presence, and we also collected rhizospheres samples to assess fungal diversity as previous work has shown the presence and diversity of fungi ( Bilgo *et al.*, 2018). For *Metarhizium* isolation, rhizosphere samples were crashed in distilled water and mosquito samples were plated in their whole on fungal selective medium containing 42 g potato-dextrose agar, 0.5 g chloramphenicol and 0.6 g cetyl trimethylammonium bromide per litter ( Bilgo *et al.*, 2018). This strain was first microscopically identified based on colony appearance, conidial shape, and conidial size ( Baró *et al.*, 2022; Bischoff *et al.*, 2009) and then confirmed by Polymerase Chain Reaction (PCR) of the Internal Transcribed Spacer (ITS), ITS1 (5’-TCCGTAGGTGAACCTGCGG-3’) and ITS4 (5’-TCCTCCGCTTATTGATATGC-3’). PCR was performed with an initial denaturation at 95°C for 10 minutes, followed by 45 cycles of 95°C for 10 seconds, 58°C for 10 seconds, and 72°C for 40 seconds of annealing. A final extension at 72°C for 5 minutes was included, and the reaction was subsequently maintained at 10°C (Kouadio *et al.*, 2018).

***Determination of fungal formulation concentration for bioassays.*** To evaluate the fungal isolates pathogenicity on *An. coluzzii*, a suspension of each isolate was prepared at a concentration of 10 ^7^ conidia/mL. This concentration allows each mosquito to be infected with an average of 211 ± 13 spores ( Bilgo, 2018). The concentration was determined using a microscope (LEICA BME) and an Improved Neubauer haemocytometer ( Baró *et al.*, 2022; Zhang *et al.*, 2020). Briefly, this technique involved suspending *Metarhizium* conidia in 0.05% Tween 80 (Polysorbate Tween® 80), followed by homogenisation using a vortex (Vortex Genie 2) for 5 min. This procedure allows the separation of conidia and ensures their uniform distribution within the solution, thereby enabling accurate enumeration for the determination of suspension concentration. The solution was then filtered through a single-layer filter sheet to separate culture medium debris from conidia. To determine the concentration, the previously obtained suspension was diluted 1:10 with 0.05% Tween 80 solution, homogenised, then 10 μL was introduced into an Improved Neubauer haemocytometer. Conidia were counted using a microscope at magnification 40X.

### Bioassays

***Initial fungal bioassay to determine the most virulent strain effective against mosquitoes.*** Approximately 100 mosquitoes (4 replicates of 25 mosquitoes each) were infected with each of the 3 fungal isolates of *Metarhizium pingshaense* (S10, S26, and S31) or a conidia-free control Tween 80 solution. For both treated and control mosquitoes, each batch of mosquitoes were anaesthetised at -20 °C for 15 seconds and then sprayed with the fungal solution or 0.05% Tween 80 solution for the controls before their recovery.

After spraying, the mosquitoes were allowed to reanimate. Then, mosquitoes were fed with 5% glucose solution and kept at 25 °C ± 2 °C and 80% ± 10% relative humidity. Mosquito mortality was monitored for 14 days after infection. Mosquitoes mortality was assessed up to 14 day as the extrinsic incubation period of *Plasmodium falciparum* is usually 10–12 days (Jefferson *et al.*, 1992; Ohm *et al.*, 2018). Dead mosquitoes were collected twice a day. Cadavers were immediately removed and individually washed for 20 seconds with 1% sodium hypochlorite, followed by sterile distilled water for 40 seconds, placed on agar, and incubated in the dark at 27°C. This step allows the elimination of other contaminants that might be present on the mosquito cuticle. Fungal growth was assessed three days later and used to confirm the success of the fungal infection ( Bilgo *et al.*, 2017).

**Figure 1.**
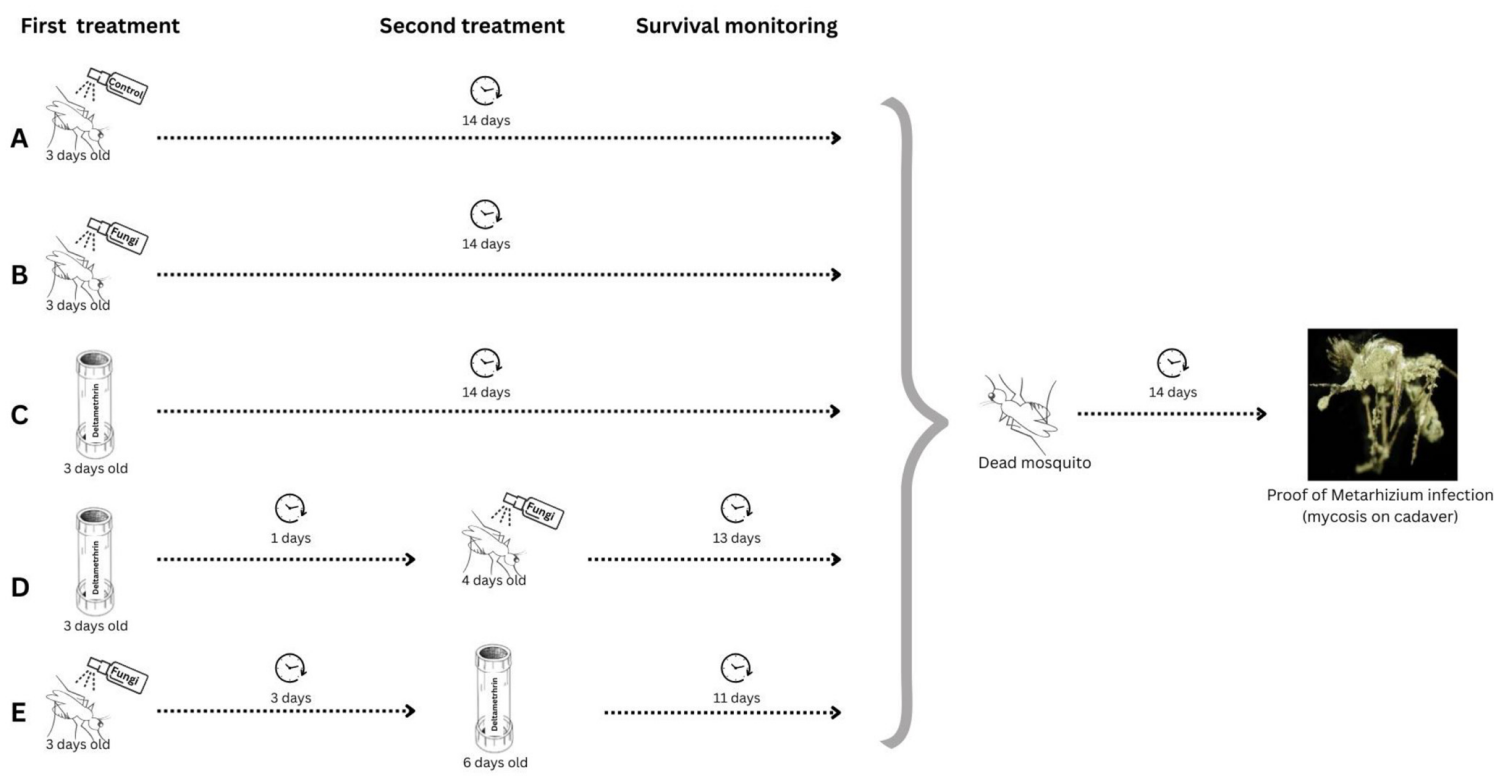
Schematic of the experimental design. This diagram illustrates the two combination sequences used to evaluate the effects of *Metarhizium pingshaense* and deltamethrin combination on insecticide resistant *Anopheles coluzzii* mosquitoes: ( **A**) control, spread with fungal free tween solution ( **B**) fungal infection only; ( **C**) Deltamethrin only exposure; ( **D**) Deltamethrin exposure followed by fungus infection; and ( **E**) fungal infection followed by deltamethrin exposure.

***Metarhizium pingshaense strains and deltamethrin compatibility test.*** Two approaches were established to assess the most virulent *M. pingshaense* and deltamethrin combination against insecticide-resistant *An. coluzzii* and compared to *M. pingshaense* infection only. Insecticide-impregnated sheets with a discriminating concentration of 0.05% deltamethrin were obtained from Vector Control Research Unit, University Sains Malaysia, Penang, Malaysia. Fungi after insecticide: mosquitoes were exposed to 0.05% deltamethrin for one hour in WHO bioassay tubes. The test involves exposing 20–25 *An. coluzzii* VKPER for 1 hour to deltamethrin (World Health Organization, 2016). After exposure, mosquitoes were transferred into to control tubes and fed on 5% glucose solution. Twenty-four hours post insecticide exposure, surviving mosquitoes were infected with *Metarhizium pingshaense* (10 ^7^ conidia/ml). Fungi before insecticide: mosquitoes were infected with fungi suspension (10 ^7^ conidia/ml), then transfer into cups and fed on 5% glucose solution. Three days later surviving mosquitoes were exposed to 0.05% deltamethrin using WHO bioassay tubes. For comparison purpose, some mosquitoes were exposed to deltamethrin only and another group was infected with *Metarhizium* only. In all approaches mosquito mortality was collected twice daily until 14 days after the first treatment and consisted of four replicates of 25 mosquitoes each. The first treatments were applied when mosquitoes were 3 days old. For the second treatment, mosquitoes were 4 days old in the deltamethrin followed by *Metarhizium* treatment, whereas they were 6 days old when exposed to deltamethrin in the *Metarhizium* followed by deltamethrin treatment. The experimental design is illustrated in Figure 1.

### Data analysis

Data were entered into Microsoft Windows Excel 2016, checked for accuracy, and imported into RStudio version 2.11.1 for data manipulation, visualisation, and statistical analysis. Mortality of mosquitoes 14 days after exposure to fungi was analysed using generalized linear models using binomial distribution, fungal strain (control, S10, S26, and S31) was included as fixed effect and replicates as random effect. Survival over several days after treatment was analysed using cox proportion hazard models using treatment (control, deltamethrin, S10, S26, and S31) and sequence (alone, fungi before deltamethrin, fungi after deltamethrin, fungi with deltamethrin) as fixed effects, and replicates as random effect. Model simplification was performed using Likelihood Ratio Tests (LRT) with p value < 0.05 as cut off.

## Results

### Native strains of *Metarhizium pingshaense* virulence against Insecticide-resistant mosquito strain *Anopheles coluzzii* VKPER

**Figure 2.**
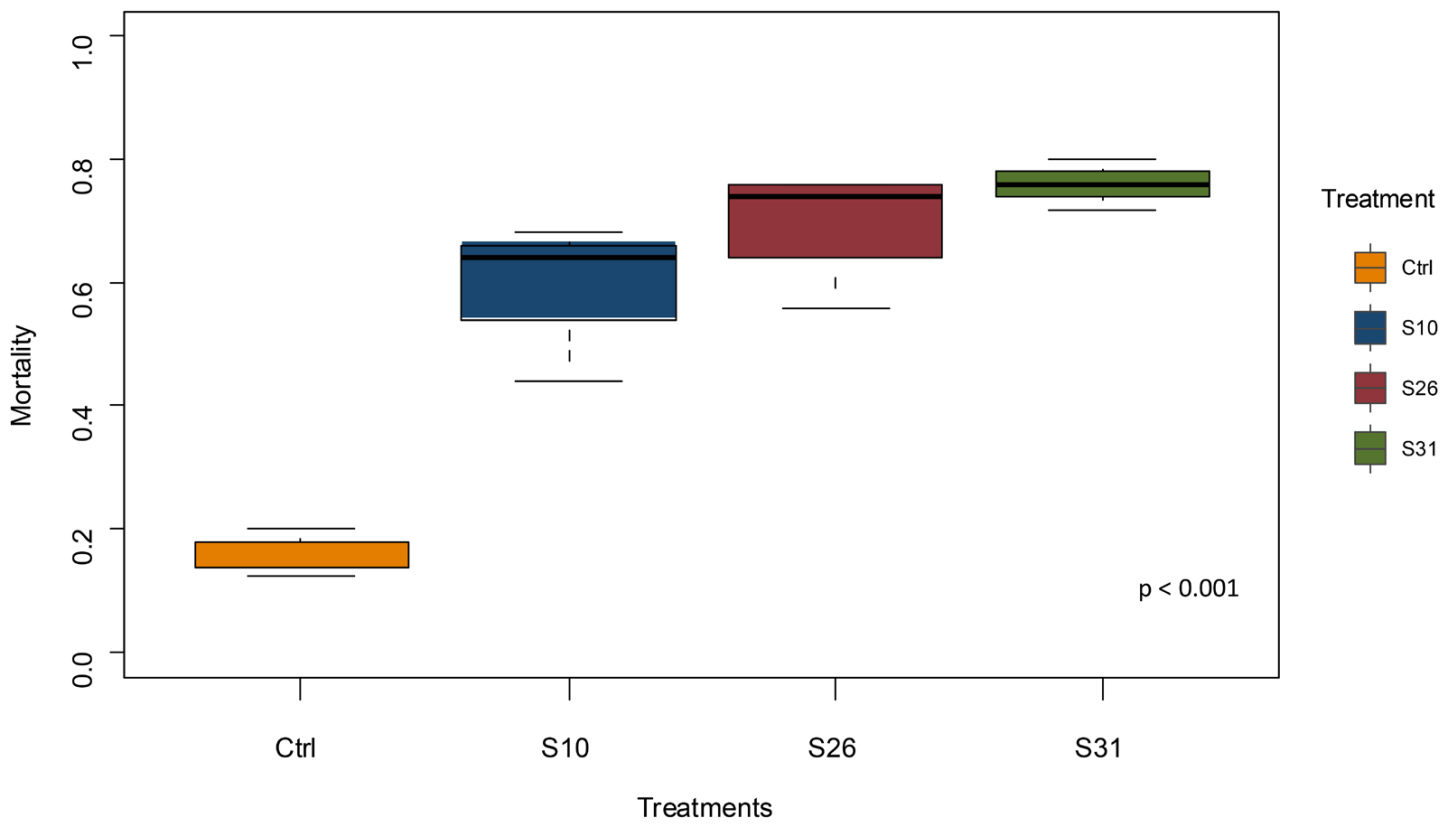
Mean percentage mortality of insecticide-resistant *An. coluzzii* VKPER infected with native *Metarhizium* strains 14 days after treatment. Three *Metarhizium* strains were used: two reference strains (S10 and S26), and a newly isolated strain (S31). Mortality was significantly higher in fungus-treated groups, regardless of the fungal strain, compared to the control group.

Adult female *Anopheles coluzzii* aged 3–5 days were exposed to a newly isolated *Metarhizium pingshaense* strain S31 and two previously isolated strains (S10 and S26) at concentration of 1 × 10 ^7 ^conidia/ml and 14-day post-infection survival compared to a control solution. All fungal treatments significantly increase mortality of mosquitoes compared to control ( Figure 2). Mosquitoes had 7.9 (95%CI: 4.112–15.75), 12.2 (95%CI: 6.31–24.96), and 16.6 (95%CI: 8.41–34.61), times the odds of dying with S10, S26 and S31 respectively, compared to control (χ ^2^ = 102.525, df = 3, p < 0.001). These results indicate that all fungal strains increased mortality, with S31 showing the highest odds ratio among the tested strains.

### Survival of mosquitoes following exposure to *M. pingshaense* and deltamethrin

**Figure 3.**
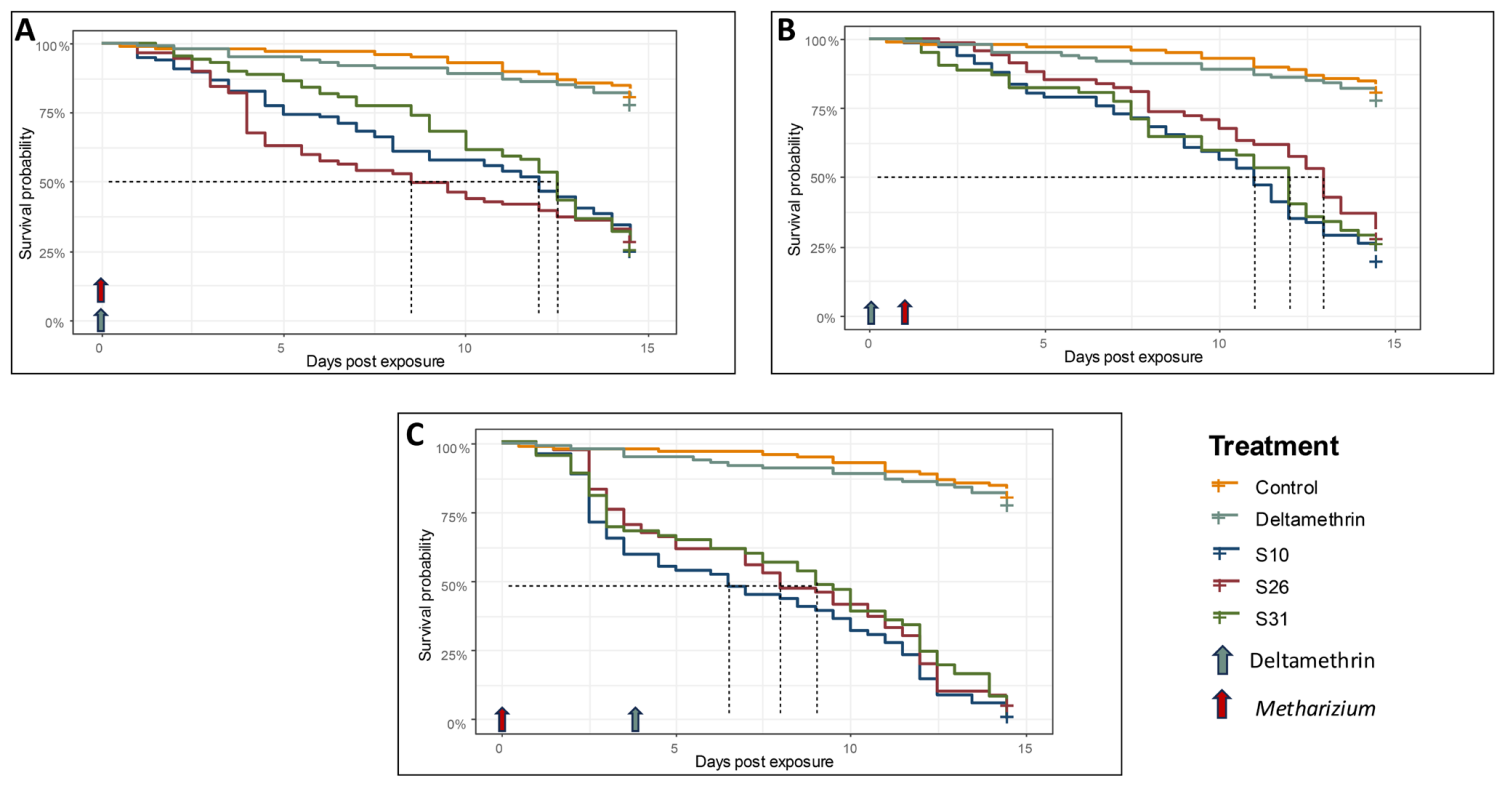
Effect of *Metarhizium* and deltamethrin on survival in insecticide-resistant *Anopheles* mosquitoes. *Metarhizium* and deltamethrin applied separately ( **A**). Deltamethrin followed by *Metarhizium* ( **B**). *Metarhizium* followed by deltamethrin ( **C**).

We then tested if these locally isolated fungal strains could increase susceptibility to insecticides in a highly resistant strain of *Anopheles coluzzii*. Cox proportional hazard analysis showed that both treatment (fungi or insecticide, χ ^2^=145.05, df=4, p < 2.2e-16) and the sequence of application (χ ^2 ^= 217.14, df = 3, p < 2.2e-16) significantly affected the mortality risk compared to the control ( Table 1). Specifically, when *Metarhizium* was applied on its own, it increased the risk of death by 6.4 (95%CI: 3.7-11.2) to 7.6 (95%CI: 4.3-13.1) fold depending on the strain; in contrast, deltamethrin effect was similar to control (Hazard ratio = 1.2, 95%CI:0.6-2.3), as expected in a highly insecticide resistant strain ( Figure 3A). When fungi were applied three days after insecticide exposure, survival analysis showed a pattern similar to that of fungi applied alone, with 6.07 (95%CI: 3.4-10.7) to 8.3 (95%CI: 4.7-14.6) time increase ( Figure 3B), as expected as this resistant colony can well tolerate deltamethrin exposure. However, when fungi were applied three days before the insecticide, we observed a significant reduction in survival, ranging from 13.1 (95%CI: 7.6-22.8) to 16.2 (95%CI: 9.3-28.1) fold increase in mortality compared to control depending on the strain; LT50 ranged from 6.8 to 8 days compared to 10 to 11.8 days when only fungi were used ( Figure 3C). This result suggests that fungi increased the susceptibility to deltamethrin in highly insecticide resistant mosquitoes and combining *Metarhizium* with chemicals can achieve greater mortality when mosquitoes are exposed to insecticide following exposure to *Metarhizium* 3 days prior. All relative statistics to the survival analysis are summarise in Table 1.

**Table 1.** Statistics of Cox proportional hazard analysis of insecticide resistant *An. coluzzii* survival treated with insecticide or fungi only, or different sequence combinations. Replicate was included as a random effect.

Treatment	Hazard Ratio (95% CI)	LT50 (days)
Control	1 (Reference)	28.38
Deltamethrin only	1.21 (CI: 0.6-2.35)	28.79
S10 only	7.53 (CI: 4.1-12.18)	11.04
S26 only	6.68 (CI: 4.37-13.17)	10.08
S31 onlys	6.77 (3.74-11.23)	11.77
S10 after Deltam	8.01 (4.56-14.07)	10.62
S26 after Deltam.	5.92 (3.35-10.45)	12.38
S31 after Deltam.	7.01 (3.95-12.43)	11.18
S10 before Deltam.	16.24 (9.37-28.13)	6.83
S26 before Deltam.	13.18 (7.6-22.85)	7.94
S31 before Deltam.	13.75 (9.91-23.88)	7.98

## Discussion

In this study we tested if locally isolated strains of entomopathogenic fungi *Metarhizium pingshaense* combined with the pyrethroid insecticide deltamethrin can overcome insecticide resistance in highly resistant malaria mosquito colony of *Anopheles coluzzii*. We found that the order of exposure of fungi and insecticide to mosquitoes is critical: infecting mosquitoes with *Metarhizium* three days before deltamethrin exposure can increase mortality up to ~13 fold higher than deltamethrin alone; LT50 of exposure to deltamethrin decreased from 28.79 days when applied alone to 6.83 days when used after S10 strain infection. In contrast, when fungi were applied after the insecticide, no major effect was observed in comparison to fungi alone.

Several physiological mechanisms may explain the synergism observed when mosquitoes were first infected with fungi and then exposed to insecticides. Previous study has shown similar results with *Metarhizium anisopliae and Beauveria bassiana* strains applied prior to permethrin exposure in a field insecticide resistant *An. gambiae*. ( Farenhorst *et al.*, 2010). Farenhorst and colleagues suggested that fungal bioactive compounds like destruxins, might interfere with enzymatic pathways involved in insecticide resistance, such as those governed by cytochrome P450 enzymes, esterases, or glutathione S-transferases. This interference could weaken the mosquito's resistance mechanisms, allowing insecticides to be more effective and thus contributing to the observed synergistic effect.( Farenhorst *et al.*, 2009). *Metarhizium* infection may increase mosquito susceptibility to insecticides through multiple mechanisms. One potential mechanism involves the disruption of cuticular integrity by the fungus, which could facilitate insecticide penetration and weaken the mosquito’s physical defenses ( Ortiz-Urquiza & Keyhani, 2013; Yahouédo *et al.*, 2017). During *Metarhizium* infection, enzymes like proteases, chitinases, and lipases are produced by the fungus. These enzymes break down proteins, chitin, and lipids in the mosquito cuticle. This joint enzymatic action might have weakened the cuticle, making mosquitoes more susceptible to insecticides and leading to mortality ( Aw & Hue, 2017). Finally, another mechanism could rely on *Metarhizium* exploiting the mosquito host energy reserves, by increasing trehalase production for fungal growth, thus impairing mosquito metabolic resistance ( Aw & Hue, 2017). Combined, cuticular, energetic, and metabolic vulnerabilities could contribute to synergistic effects with deltamethrin. 

The survival of mosquitoes infected with *Metarhizium pingshaense* one day after deltamethrin exposure was similar to the survival of *Metarhizium pingshaense* applied alone. Although most of the insecticide effects of deltamethrin on mosquitoes are immediate and rapid within 24-hours post exposure, some authors have shown that exposure to the insecticide can affect mosquito survival up to 14 days after exposure ( Viana *et al.*, 2016). However, this delayed effect of deltamethrin does not appear to be sufficiently important to have caused a statistically significant additive effect to that of *Metarhizium* alone. 

The virulence results confirmed the pathogenicity of the native isolates of *Metarhizium spp.* from Burkina Faso against the major malaria vector, *An. coluzzii* as reported by Bilgo and colleagues ( Bilgo *et al.*, 2018) including a new isolate *Metarhizium* S31. The similarity between the mortalities of the three tested isolates could be due to their geographical proximity or the organisms from which they were isolated. Indeed, isolates S26 and S31 were isolated from mosquitoes of the genus *Anopheles* and plant roots, respectively, in the village of Bana. Like S26, isolate S10 also came from an *Anopheles* mosquito collected from an uninhabited house in Soumousso village ( Bilgo *et al.*, 2018). However, isolates S10 and S31 were distant both geographically and in terms of their origin. At the intermediate concentration of 10 ^7^ conidia/ml, no isolate reached the 80% mortality threshold set by the World Health Organization Pesticide Evaluation System (WHOPES) for effective insect control with insecticides ( World Health Organization, 2016b). Bilgo and colleagues reported a mortality rate > 80% in 14 days at the same concentration ( Bilgo *et al.*, 2018). Additionally, a study conducted in Benin by Howard and colleagues reported similar results ( Howard *et al.*, 2010). It’s important to specify that the 80% mortality in 24h threshold has been set for chemical insecticides which are fast acting for a statistically significant epidemiological impact. However, it might not be adapted for biological insecticide evaluation which act slowly in which case epidemiological impact might be visible in the long run.

The use of native fungal strains in vector control strategies offers several advantages. Native isolates are often better adapted to local environmental conditions than exotic strains, potentially enhancing their persistence and efficacy in the field. Furthermore, their use poses a lower ecological risk compared to introducing non-native organisms. From a social and regulatory perspective, native strains are also more likely to be accepted by end users and stakeholders than exotic or genetically modified ones, which often face resistance due to ethical concerns and regulatory constraints.

This finding could lead to a new IPM strategy for malaria vector control using *Metarhizium pingshaense* as an IRS product in conjunction with the use of LLINs. The use of metarhizium-impregnated fabrics placed in strategic mosquito resting places could also be considered while maintaining the current vector control strategies. Female mosquitoes typically take a blood meal every 2–3 days until they die ( Killeen, 2020), this timeframe that closely aligns with the fungal incubation period in mosquitoes. When LLINs are used alongside *Metarhizium* applied to resting surfaces, mosquitoes may first become infected with the fungus during their post-blood meal resting period. By the time they seek their next blood meal, they would then encounter the insecticide-treated LLIN. Given the efficacy of the combination fungi followed by insecticide in laboratory conditions it would be interesting to evaluate in a scenario closer to field delivery formats in semi field experiment in malaria sphere.

## Conclusion

Native strains *Metarhizium pingshaense* combined with deltamethrin showed a synergistic effect on insecticide resistant *An. coluzzii* VKPER. However, this synergy is highly dependent on the order of application, with *Metarhizium* infection prior to deltamethrin exposure being the most effective sequence. Both *Metarhizium* and deltamethrin align well with IVM approach, which is strongly recommended by the WHO for managing insecticide resistance in malaria vectors. However, it is necessary to better understand the synergy observed in this experiment by evaluating insecticide resistance related genes expression during *Metarhizium* infection. It is also appropriate to evaluate these combinations in a semi-field condition.

## Ethics and consent

Ethical approval and consent were not required.

## Data Availability

Repository: Entomopathogenic fungi *Metarhizium pingshaense* increases susceptibility to insecticides in highly resistant malaria mosquitoes *Anopheles coluzzii* https://doi.org/10.5525/gla.researchdata.1636 This project contains the following underlying data: ltdata.csv [Timeline mosquito mortality dataset] Mortality.csv [Fungi virulence against *Anopheles coluzzii*] Survival_analysis.csv [Mosquito survival dataset] LT50.R [R code for mosquito LT50 analysis] Mortality.R [Fungi virulence analysis R code] Survival_analysis.R [Mosquitoes survivasl analysis R code] Data are available under the terms of the the Creative Commons Attribution Licence https://creativecommons.org/
