# Supplementary material for: Entomopathogenic fungi Metarhizium pingshaense increases susceptibility to insecticides in highly resistant malaria mosquitoes Anopheles coluzzii
Source: Wellcome Open Res. 2025 May 22;9:290. Originally published 2024 May 22. [Version 3] doi: 10.12688/wellcomeopenres.21238.3 (PMC12107690; doi:10.12688/wellcomeopenres.21238.3)
Supplement: Peer review history

# Reviewer report: Reviewer response for version 3

**DOI:** 10.21956/wellcomeopenres.26744.r123974

**Published:** 2025-05-24

**Authors:** Ambrose Oruni

**Affiliations:** 1 Liverpool School of Tropical Medicine, Liverpool, UK

No new comments.

Is the work clearly and accurately presented and does it cite the current literature?

Partly

If applicable, is the statistical analysis and its interpretation appropriate?

Partly

Are all the source data underlying the results available to ensure full reproducibility?

Yes

Is the study design appropriate and is the work technically sound?

Partly

Are the conclusions drawn adequately supported by the results?

No

Are sufficient details of methods and analysis provided to allow replication by others?

Partly

Reviewer Expertise:

Vector Biology, Molecular Genetics and Field trials and operational research.

I confirm that I have read this submission and believe that I have an appropriate level of expertise to confirm that it is of an acceptable scientific standard.

# Reviewer report: Reviewer response for version 2

**DOI:** 10.21956/wellcomeopenres.26006.r119383

**Published:** 2025-05-12

**Authors:** Ambrose Oruni

**Affiliations:** 1 Liverpool School of Tropical Medicine, Liverpool, UK

The authors addressed most of my concerns. 

 As a I final edit, if they could please upload another Figure 2 without the underlined words.

 Otherwise, I'm happy with the revision.

Is the work clearly and accurately presented and does it cite the current literature?

Partly

If applicable, is the statistical analysis and its interpretation appropriate?

Partly

Are all the source data underlying the results available to ensure full reproducibility?

Yes

Is the study design appropriate and is the work technically sound?

Partly

Are the conclusions drawn adequately supported by the results?

No

Are sufficient details of methods and analysis provided to allow replication by others?

Partly

Reviewer Expertise:

Vector Biology, Molecular Genetics and Field trials and operational research.

I confirm that I have read this submission and believe that I have an appropriate level of expertise to confirm that it is of an acceptable scientific standard.

# Reviewer report: Reviewer response for version 2

**DOI:** 10.21956/wellcomeopenres.26006.r119381

**Published:** 2025-05-09

**Authors:** Shüné Oliver

**Affiliations:** 1 Division of the National Health Laboratory Service, National Institute for Communicable Diseases, Johannesburg, South Africa; 2 University of the Witwatersrand Johannesburg, Johannesburg, Gauteng, South Africa

I am satisfied with the changes made to the document. The authors have addressed my comments.

Is the work clearly and accurately presented and does it cite the current literature?

Yes

If applicable, is the statistical analysis and its interpretation appropriate?

Yes

Are all the source data underlying the results available to ensure full reproducibility?

Yes

Is the study design appropriate and is the work technically sound?

Yes

Are the conclusions drawn adequately supported by the results?

Yes

Are sufficient details of methods and analysis provided to allow replication by others?

Partly

Reviewer Expertise:

Malaria vector biology, Insecticide resistance.

I confirm that I have read this submission and believe that I have an appropriate level of expertise to confirm that it is of an acceptable scientific standard.

# Reviewer report: Reviewer response for version 2

**DOI:** 10.21956/wellcomeopenres.26006.r120192

**Published:** 2025-03-25

**Authors:** Maria Kaiser

**Affiliations:** 1 National Institute for Communicable Diseases., Johannesburg, South Africa

**Review: **Entomopathogenic fungi *Metarhizium pingshaense *increases susceptibility to insecticides in highly resistant malaria mosquitoes *Anopheles coluzzii*

 Three *Metarhizium pingshaense* isolates (2 previously isolated and 1 newly isolated strain) from Burkina Faso were tested for virulence against the An. coluzzii VKPRer. Similar/ slightly different mortality was achieved in the mosquitoes exposed to the three isolates as measured after 14 days

 All three isolates were then tested for their efficacy in increasing mosquito mortality when combined with the insecticide deltamethrin (and compared to Metarhizium alone) in the following sequences

  

1. Deltamethrin followed by Metarhizium 1 day later
2. Metarhizium followed by deltamethrin 3 days later
3. Deltamethrin and Metarhizium simultaneously

Controls

 Not exposed

 Exposed to deltamethrin

 These were tested against a highly pyrethroid resistant *Anopehles coluzzii *colony (VKPRer)

 The sequence of exposure to the biocontrol agent/s and deltamethrin insecticide significantly affected mosquito mortality compared to Metarhizium exposure alone: Metarhizium exposure followed 3 days later by deltamethrin exposure significantly reduced the mosquito lifespan (synergistic effect) while deltamethin exposure followed one day later by Metharhizium exposure had no effect and the simultaneous exposure to deltamethrin and Metarhizium produced antogonistic results (less mortality that when mosquitoes were exposed the Metarhizium alone). 

**Comments:**

**In conclusion section of Abstract:**

 Instead of saying …synergically improve mosquito control in the laboratory it might be more accurate to say something like… synergically increased mosquito susceptibility to the insecticide in the laboratory

**keywords:**

 Some keywords (species names) need to be italisized

**In background:**

 3 ^rd^ sentence should be transmission potential through vector control, not throughout

 Since the early 2 000s, i nterventions… instead of 2000 and intervention

 ‘Co-treated ITNs with Piperonyl Butoxide (PBO) have already been rolled out to act as synergist for chemical insecticide.’ This sentence could read better- E.g. ITNs co-treated with Piperonyl Butoxide (PBO) synergist have already been rolled out….

 ‘difficulties to develop new class of insecticide’ could read better e.g… and the difficulties and long timeframes often required to develop new classes of insecticides limi ts…

 ‘address insecticide resistance in malaria vector control still crucial’ should be is crucial or is still crucial.

 Aw and Hue- is not an appropriate reference for human infection with Metarhizium- suggest removing it. Nourrisson et al is appropriate.

 ‘Thus, even infected, some mosquitoes can survive long enough to transmit Plasmodium to humans’ It might be a good idea so show here what the incubation time is (time until mosquitoes are able to transmit malaria) here instead of only in the discussion

 ‘on their insecticide susceptibility. However, when the infection reached its lethal time (3 days post-infection), a subsequent deltamethrin exposure caused higher mortality (Farenhorst et al., 2010).’ This doesn’t capture Farenhorst et al’s findings please check…Farenhorst et al reported, Met followed by perm 3 days later had no significant effect- also see they used permethrin and not deltamethrin

 ‘it is conceivable that a mosquito may be exposed to the insecticide through the LLIN prior to being infected with the fungus on the treated surface. An additional scenario involves combining both elements on single substrates such as walls or bednets.’ …. these are the most conceivable options given fungal application on surfaces on which adult mosquitoes would rest- what are options that could expose mosquitoes to fungi before insecticide contact- that is an important question for the work you have presented here (probably more for discussion section)

 ‘We found M. pingshaense infected mosquitoes have improved sensitivity to deltamethrin.’ Perhaps specify that this was dependent on the order and timing of application of each product- but also not needed to specify what was found in the background section, as this information appears later

**Methods:**

**Mosquito strains**

 ‘Only females of 3 to 5 days old, not blood fed were used and maintained in the laboratory under optimal conditions at 25 ± 2 °C, 80% ± 10% relative humidity, and a photoperiod of 12:12 (L:D) at Institut de Recherche en Sciences de la Santé (IRSS).’ Rather first describe how mosquitoes were maintained. Then that 3-5 day old females were used for experiments.

**Fungal stains**

 ‘We collected mosquitoes from the house, woodpile and from rhizosphere as our previous work showed presence and diversity of fungi’ do you mean mosquitoes or fungal samples?  or did you collect mosquitoes from the house and wood pile for testing of fungal samples and also collect rhizosphere samples to test for fungus?.. this is a bit confusing

 ‘and then confirmed by Polymerase Chain Reaction (PCR) of the Internal Transcribed Spacer (ITS), ITS1 (5’-TCCGTAGGTGAACCTGCGG-3’) and ITS4 (5’- TCCTCCGCTTATTGATATGC-3’). PCR was performed with an initial denaturation at 95°C for 10 minutes, followed by 45 cycles of 95°C for 10 seconds, 58°C for 10 seconds, and 72°C for 40 seconds of annealing. A final extension at 72°C for 5 minutes was included, and the reaction was subsequently maintained at 10°C’… are these your own developed methods or previously published- if previously published please add reference.

***Determinations of fungal formulation concentrations for bioassays***

 ‘Insecticide-impregnated sheets with a discriminating concentration of 0.05% deltamethrin were obtained from Vector Control Research Unit, University Sains Malaysia, Penang, Malaysia.’ Should be moved to underneath bioassays section

***Metarhizium pingshaense strains and deltamethrin compatibility test***

 ‘Simultaneous approach: a fungal suspension(10 ^7 ^conidia/ml) was sprayed onto a 0.05% deltamethrin sheet and allowed to dry for 30 minutes; mosquitoes were then exposed to the sheet using a WHO insecticide susceptibility test tubes for one hour (World Health Organization, 2016b).’ could this have coated the insecticide and made it less likely that the mosquitoes contacted it? maybe something to discuss as well as the different application method for the fungus used here

 ‘In all approaches mosquito mortality was collected twice daily until 14 days after the second treatment and consisted of four replicates of 25 mosquitoes each.’ for each of these methods please state how old the mosquitoes were at final exposure? I.e was this done at the same age for each group?  i/e were mosquitoes collected 14 days into the survival experiments all the same age regardless of treatment?

**Results:**

 Native strains of *Metarhizium pingshaense* virulence against Insecticide-resistant mosquito line *Anopheles coluzzii*

 ‘Mosquitoes had 7.9 (95%CI: 4.112-15.75), 12.2 (95%CI:6.31-24.96), and 16.6 (95%CI: 8.41-34.61), times the odds of dying with S10, S26 and S31 respectively, compared to control(χ2 = 102.525, df = 3, p < 0.001), suggesting that the newly isolated strain S31 is the most virulent of all.’ Do you have pairwise comparisons (p-values and indictors) to support S31 is most virulent statistically?

                Survival of mosquitoes following exposure to M. pingshaense and deltamethrin

 ‘This result suggests that fungi increased the susceptibility to deltamethrin in highly insecticide resistant mosquitoes and combining Metarhizium with chemicals can achieve greater mortality.’ .. suggest adding when mosquitoes are exposed to insecticide following exposure to Metarhizium 3 days prior (or similar).. especially as the next sentence speaks about the co-formulation being less effective that Metarhizium alone, and also goes on to mention antagonist effects

**Discussion:**

 ‘The survival of mosquitoes infected with *Metarhizium pingshaense* three days after deltamethrin exposure was similar to the survival of *Metarhizium pingshaense* applied alone.’ …Do you mean one day after

 'In contrast, an antagonistic effect was observed between *Metarhizium* and deltamethrin were combined simultaneously.'.. do you mean when

  ‘by Bilgo et al. (Bilgo et al., 2018) including a newly isolate S31.’ Would read better as .. a newly isolated strain of M. p , S31… or a new isolate, S31

 ‘At the intermediate concentration of 107 conidia/ml, no isolate reached the 80% mortality threshold set by the World Health Organization Pesticide Evaluation System (WHOPES) for effective insect control with insecticides (World Health Organization, 2016b). However, this threshold may not directly apply to biological agents. Bilgo et al. reported a mortality rate > 80% in 14 days at the same concentration (Bilgo et al., 2018). Additionally, a study conducted in Benin by Howard et al reported similar results (Howard et al., 2010’ ….I suspect the point that is being made here is not coming across clearly. The sentences ‘However, this threshold may not directly apply to biological agents.’ And ‘Bilgo et al. reported a mortality rate > 80% in 14 days at the same concentration (Bilgo et al., 2018). Additionally, a study conducted in Benin by Howard et al reported similar results (Howard et al., 2010’ are not linking well. Explain the link here- why is it important or relevant that mortality is >80% at 14 days. Perhaps also mention the time it took to reach mortality in these 2 referenced studies- it was faster that 14 days but the biological agents used were different to what you tested and one was modified with a spider neurotoxin

 ‘This finding could lead to a new IPM strategy for malaria vector control using Metarhizium pingshaense as an IRS product in conjunction with the use of LLINs. The use of Metarhizium impregnated fabrics placed in strategic mosquito resting places could also be considered while maintaining the current vector control strategies. Given the efficacy of the combination fungi followed by insecticide in the lab, it would be interesting to evaluate in a scenario closer to field delivery formats in semi field experiment in malaria sphere.’ … this section deserves further discussion given the order in which the specific biocontrol agent/s and insecticide need to be applied. Also considering the most conceivable options for delivery, which were mentioned in the introduction.

**Conclusion:**

 ‘Combining Metarhizium pingshaense with deltamethrin may enhance its efficacy against insecticide-resistant mosquitoes, creating a synergistic effect.’ … No emphasis is placed on the order of exposure here but that was an important finding from the work presented as other orders resulted in similar effect to Met alone or even worse than Met alone- it is important to emphasize that the order of application is critical here

 It might be a good idea to also include that the met strains used in the tests were locally obtained and that a new strain was identified.

 'This study provides a basis for the successful integrated use of entomopathogenic fungi and chemical insecticides' ...this statement overreaches, please consider revising

**Figures/ tables:**

 ‘Figure 1. Mortality repartition of insecticide-resistant An. coluzzi VKPER infected with native Metarhizium strains, 14 days after treatment. Three Metarhizium strains were used: two reference strains (810 and 826), and a newly i solates strain (831). virulence against the insecticid-resistant, malaria vector Anopheles coluzzi VKPER.’….

 the last sentence of this legend ends abruptly do you mean i s shown

8 has been included here where it should be S for the strain names,

 check underlined words for spelling/ grammar.

 Instead of repartition it would be more informative to indicate if this is mean percentage mortality / proportional mortality,

 also please indicate where there are, or are no, significant differences between pairs. Either here in the figure/ table or this can be included in the text

 This legend does not make sense: ‘Figure 2. Effect of Metarhizium and deltamethrin on survival in insecticide-resistant Anopheles mosquitoes. Metarhizium applictaion alone (A). Metarhizium application before deltamethrin (B). Metarhizium application after deltamethrin (C). Co-formulation of Metarhizium and deltamethrin (D).’

 For B do you mean deltamethrin followed by Metarhizium?

 For C do you mean Metarhizium followed by deltamethrin? (Panles B and C maybe switched position?)

 Having both arrows in Panel A of Figure 2 seems like mosquitoes were exposed to deltamethrin immediately followed by Metarhizium but this should be the panel for Metarhizium alone so should have only the Metarhizium arrow

**General:**

- Please stick to mosquito strain or mosquito line and avoid using these interchangeably
- Adding P values to figure 1 has already been requested by another reviewer as well and is not in this version- is this the updated version?
- I also suggest adding to the discussion the use of native rather vs exotic strains as requested previously by other reviewer
- Please discuss if/how the two different fungal applications used in this work might have affected the results.

Is the work clearly and accurately presented and does it cite the current literature?

Partly

If applicable, is the statistical analysis and its interpretation appropriate?

Partly

Are all the source data underlying the results available to ensure full reproducibility?

Yes

Is the study design appropriate and is the work technically sound?

Partly

Are the conclusions drawn adequately supported by the results?

Partly

Are sufficient details of methods and analysis provided to allow replication by others?

Partly

Reviewer Expertise:

Malaria vector biology and ecology, malaria vector surveillance, malaria vector control, insecticide resistance

I confirm that I have read this submission and believe that I have an appropriate level of expertise to confirm that it is of an acceptable scientific standard, however I have significant reservations, as outlined above.

## References

[1] : Transgenic Metarhizium pingshaense synergistically ameliorates pyrethroid-resistance in wild-caught, malaria-vector mosquitoes. *PLoS One* .2018;13(9) : 10.1371/journal.pone.0203529 e0203529 10.1371/journal.pone.0203529 30192847 PMC6128571

[2] : Synergy in Efficacy of Fungal Entomopathogens and Permethrin against West African Insecticide-Resistant Anopheles gambiae Mosquitoes. *PLoS ONE* .2010;5(8) : 10.1371/journal.pone.0012081 10.1371/journal.pone.0012081 PMC292033520711409

[3] : Pyrethroid resistance in Anopheles gambiae leads to increased susceptibility to the entomopathogenic fungi Metarhizium anisopliae and Beauveria bassiana. *Malar J* .2010;9: 10.1186/1475-2875-9-168 168 10.1186/1475-2875-9-168 20553597 PMC2898789

[4] : Species of Metarhizium anisopliae complex implicated in human infections: retrospective sequencing study. *Clin Microbiol Infect* .2017;23(12) : 10.1016/j.cmi.2017.05.001 994-999 10.1016/j.cmi.2017.05.001 28487164

# Reviewer report: Reviewer response for version 2

**DOI:** 10.21956/wellcomeopenres.26006.r119382

**Published:** 2025-03-12

**Authors:** Tullu Bukhari

**Affiliations:** 1 International Centre of Insect Physiology and Ecology, Nairobi, Kenya

The major issue remains the methodology of the compatibility test.

Is the work clearly and accurately presented and does it cite the current literature?

Partly

If applicable, is the statistical analysis and its interpretation appropriate?

Partly

Are all the source data underlying the results available to ensure full reproducibility?

No

Is the study design appropriate and is the work technically sound?

Partly

Are the conclusions drawn adequately supported by the results?

Partly

Are sufficient details of methods and analysis provided to allow replication by others?

Partly

Reviewer Expertise:

Entomopathogenic fungus for vector control, Lethal and sub-lethal effects of pathogens and symbionts, Symbiont-based transmission-blocking strategy, Genetics and genomics of vector immune responses to microbes.

I confirm that I have read this submission and believe that I have an appropriate level of expertise to confirm that it is of an acceptable scientific standard, however I have significant reservations, as outlined above.

# Reviewer report: Reviewer response for version 1

**DOI:** 10.21956/wellcomeopenres.23487.r93091

**Published:** 2024-09-06

**Authors:** Tullu Bukhari

**Affiliations:** 1 International Centre of Insect Physiology and Ecology, Nairobi, Kenya

Microbes have conventionally been used or proposed to control disease vector populations, as an alternative to insecticides.  However, delayed mortality, variation in production batches, loss of virulence in the natural environment, and cost have influenced the uptake of these microbes. This study adds to the growing evidence that microbes have sub-lethal effects that can also contribute to disease control in addition to the reduction of vector populations. Understanding the sub-lethal effects can inform how microbes can be used to complement core disease control tools. Please see my comments below. Note that this is in addition to general comments from the first reviewer. I support the comment that there is a need to improve the writing to make it clear and concise and ensure there are no spelling and grammatical mistakes.

**Major comments:**

1. The authors report a new strain (S31). However, no data or analysis (sequencing, ANI comparison) is provided to support this claim.
2. The mosquitoes were exposed to fungus and the insecticide in three sequences. In the sequence where mosquitoes are simultaneously exposed to insecticide and fungus, the fungus spores are sprayed on the insecticide-treated surface and the mosquitoes are exposed to the surface. While in the fungi-only or fungi-first treatments, the mosquitoes are sprayed directly. This methodology does not ensure that mosquitoes acquire the same number of spores; even if the spores’ concentration was similar, mosquitoes probably picked fewer spores in the simultaneous exposure. The comparison therefore is not justified.
3. Spraying mosquitoes directly does not reflect what will happen in natural settings. In a natural setting, mosquitoes will most likely pick the spores by landing/rubbing against treated surfaces, hence fewer spores will be picked.

**Minor comments:**

1. Abstract: Results: Remove “Therefore”.
2. Abstract: Conclusion: Fungus name needs to be in Italics.
3. Heading Plain language summary: “Plaine” not “plan” .
4. Plain language summary: Please rephrase “Mosquito control products **made from** a type”; the products are fungus spores and so the fungus itself, so the statement “made from fungus” is not correct.
5. Background: first paragraph: “ we assisted in a stable….”. what does “we” mean?
6. Background: second paragraph: “Although are useful in some population, but might be less effective in other populations that are very strongly resistant”. Is this a human or mosquito population?
7. Background: second paragraph: “….address insecticide resistance in malaria vector control still crucial.”  Add “is” before “still crucial.”
8. Background: third paragraph: Integrated Vector Management is the term generally used. The reference 18 does not support the statement. The statement is general and there are better references for this.
9. Background: sixth paragraph: Remove “In this study”
10. Methods: The terms “strains”, “colony”, “line” and “isolate” are used interchangeably both for mosquitoes and fungus. Please choose one with justification and be consistent.
11. Methods: Bioassay: “….14 days to determine the transmission potential of mosquitoes after *Metarhizium*…”. The transmission potential was not determined. Rephrase.
12. Result section: Please remove the methodology from the result sections to avoid repetition.
13. Data analysis: Why use two methods: GLM and survival analysis? Also for cox regression model would be more insightful if the fungus strain is used as a factor.
14. Please report the 95% CI for the Hazard ratios. Also, can HR values be interpreted as “synergism”. There are methods to calculate synergism including one used in the reference cited in the manuscript (Farenhorst, 2010).
15. Figure 2 separates what needs to be compared. My suggestion is to separate the graphs based on the fungus strains and each graph allows the comparison of fungus only, fungus first, Deltamethrin first, and simultaneous exposure.
16. Conclusion: “ *Metarhizium pingshaense* could be used as an alternative to replace chemical insecticides…” This study does not support the use of fungus to replace chemical insecticides.

Is the work clearly and accurately presented and does it cite the current literature?

Partly

If applicable, is the statistical analysis and its interpretation appropriate?

Partly

Are all the source data underlying the results available to ensure full reproducibility?

No

Is the study design appropriate and is the work technically sound?

Partly

Are the conclusions drawn adequately supported by the results?

Partly

Are sufficient details of methods and analysis provided to allow replication by others?

Partly

Reviewer Expertise:

Entomopathogenic fungus for vector control, Lethal and sub-lethal effects of pathogens and symbionts, Symbiont-based transmission-blocking strategy, Genetics and genomics of vector immune responses to microbes.

I confirm that I have read this submission and believe that I have an appropriate level of expertise to confirm that it is of an acceptable scientific standard, however I have significant reservations, as outlined above.

# Response

**Published:** 2024-12-13

**Authors:** Etienne Bilgo

**Affiliations:** Public Heath and Biomedical, Institut de Recherche en Sciences de la Santé, Bobo Dioulasso, Houet, Burkina Faso

**Reviewer** **2** **Overall comments:**

- This manuscript has the potential to be a very good piece of work. However, the authors need to carefully address the comments below and add additional work if possible. The science is there.
- The paper requires a major revision of the writing style. A lot of the sentences including figure legends require reconstruction to correct the grammar, spelling mistakes, flow and the use of the right words and also remove redundancy.

**Introduction:**

**Specific comments:** Wouldn’t it be better to introduce the disease first and vector/malaria control? Thank you for your feedback. We appreciate your suggestion to introduce the disease before discussing the vector and malaria control measures.

**Response: **In response, we have revised the introduction to context to malaria, including its significance and impact on public health and control strategies as well. *The new text was added in lines 62-65*. 

 I don’t think the language used here i.e. “we assisted …” is appropriate. Thank you for your observation regarding the language used in this section.

**Response: **We have revised this portion to adopt a more objective and formal tone, ensuring that it aligns with the overall style of the article. *Line 65-67*.

 Reference 2 is WHO malaria report 2015, please update to the current. Thank you for pointing out the need to update Reference 2.

**Response: **We have revised the citation to include the most recent WHO malaria report (Word malaria report, 2023), ensuring that the information is current and relevant.

 I think your references are misplaced, Bhatt et al talked about “averted malaria cases” not “reduction” … The 60% paragraph should have Bhatt et al reference not WHO…please revise. **Response: **Thank you for your insightful feedback regarding the references. We revised the citations to accurately reflect Bhatt *et al.*’s discussion of “averted malaria cases” instead of “reduction.”

 You mention that “…. early 2000 have led to impressive advances in malaria control….” What are these advances? Briefly state them.

**Response: **Thank you for your suggestion to clarify the advances in malaria control since the early 2000s. We revised the text to briefly outline key advancements, such as the development and distribution of insecticide-treated nets, improved diagnostic tools, the rollout of artemisinin-based combination therapies, and increased funding for malaria programs. This is now in lines 69-71.

 In this statement; “The spread of insecticide resistance in malaria vectors has led to a drop in the effectiveness of these tools, resulting in a resurgence of the disease since 2015” …this is not entirely correct. Insecticide resistance is part but there could be other factors. Please revise

**Response: **Thank you for your feedback regarding the statement on insecticide resistance and its role in the resurgence of malaria. We recognize that while insecticide resistance is a significant factor, other elements such as changes in environmental conditions, socioeconomic factors, and disruptions in health services also contribute to the resurgence. We revised the statement to reflect a more nuanced understanding of the various factors involved, in lines 77-79

 In this statement, “…with 19 billion ITNs supplied to the region from 2004 to 2019…” It should be “approximately or over 19 billion”

**Response: **Thank you for your suggestion to clarify the figure regarding insecticide-treated nets (ITNs). We revised the statement to specify "approximately 19 billion ITNs supplied to the region from 2004 to 2019".

  In this statement, “…. development of strategies to avert the selection of resistance..” I think using “strategies to reverse insecticide resistance…” sounds better because it’s probably impossible to avert it now.

**Response: **Thank you for your insightful suggestion regarding the phrasing in that statement. We agree that using "strategies to reverse insecticide resistance" more accurately reflects the current situation and challenges in malaria control. We revised the text accordingly to incorporate this more precise language.

   In this statement; “Although are useful in some population, but might be less effective in other populations that are very strongly resistant ..” you could replace with; “Although PBO nets are still effective in some mosquito populations, they show significant reduction in efficacy in other populations that are highly resistant..” with references.

**Response: **Thank you for your suggestion to improve clarity in this statement. We revised it to read: “Although PBO nets are still effective in some mosquito populations, they show significant reduction in efficacy in other populations that are highly resistant.”

 In this statement, “…develop new class of insecticide limit the practical options for simple chemical-based insecticide resistance management.” Why simple ? Plus, i expected you to reference more studies reporting cross resistance here. I thought the reference on Fungal entomopathogens should come in later.

**Response: **Thank you for your valuable feedback. We clarified the language in the statement to remove the term "simple," which may not accurately reflect the complexity of insecticide resistance management. We revised it to: "the development of new classes of insecticides limits the practical options for effective chemical-based insecticide resistance management." Additionally, we included more references that report on cross-resistance, and we ensured that the reference on entomopathogenic fungi is introduced in a more appropriate context later in the discussion.

  In this statement, “One solution has been identified in the integrated vector control (IVC), which is a combination of different tools and strategies for controlling a single vector”.  IVC combines different malaria control strategies, so your statement is confusing. Please state the one solution you are referring to, otherwise, revise the statement.

**Response: **Thank you for your feedback regarding the clarity of the statement on integrated vector control (IVC). We have revised it to specify the one solution being referred to. Revised statement: “One proposed solution is integrated vector control (IVC), which combines various malaria control strategies and tools to manage malaria transmission effectively.” This revision clarifies that IVC is a multifaceted approach rather than a single tool.

  In this statement, “Entomopathogenic fungi are probably the most common natural insect pathogens…” Are you sure they are? If so, then support the statement with some studies and evidence. Also briefly mention what other pathogens are there.

**Response: **Thank you for your important question regarding the statement about entomopathogenic fungi. While entomopathogenic fungi are indeed prevalent natural insect pathogens, we revised the statement to clarify this and support it with evidence. Revised statement: “Entomopathogenic fungi are among the most common natural insect pathogens, playing a significant role in the regulation of insect populations. Studies have shown that these fungi can effectively infect and kill various insect species (e.g. (Digvijay Singh et al., 2017; Wang et al., 2016). Other notable natural insect pathogens include bacteria (e.g., *Bacillus thuringiensis*), viruses (e.g., nucleopolyhedroviruses), and nematodes (e.g., *Steinernema* spp.), which can also contribute to biological control of insect pests.”

 In this statement, “…development as they do not need to be ingested, in contrast to bacteria and viruses (put full stop here). Indeed….”

**Response: **Thank you for your suggestion to improve the clarity of that statement. We revised it to include a full stop for better readability.

  Plus, how do the fungi occur in nature? How do they infect the mosquitoes, is it vertically, horizontally or both? What is the rate of spread in population once infected? All this information is missing

**Response: **Thank you for your valuable feedback regarding the need for more information on entomopathogenic fungi. We revised the text to include details on their natural occurrence, modes of infection, and rates of spread in mosquito populations.

 Could you please give more information about the biology of this Metarhizium spp? **Response: **Thank you for your request for more information about the biology of *Metarhizium* spp. We expanded on this in the text to provide a clearer understanding of their characteristics and life cycle.

 In this statement, “Specifically, Metarhizium requires at least 3 days to infect mosquitoes, mortalities occur within 7 days, and certain mosquitoes survive even 14 days post-infection…” If it is less effective and act slower with mosquitoes surviving even up to 14 days inoculation period, how is this a good control method for malaria?

 Maybe you could explain these scenarios where mosquitoes survive longer and why? Is it a common phenomenon that most mosquitoes survive longer and which ones do?

**Response: **Thank you for your insightful comment regarding the efficacy of Metarhizium spp. in controlling malaria-carrying mosquitoes. While it is true that Metarhizium requires a minimum of three days for infection and that some mosquitoes may survive beyond 14 days post-infection, several factors can contribute to its effectiveness as a biocontrol agent. Firstly, the effectiveness of Metarhizium is context-dependent. Environmental conditions such as humidity, temperature, and the mosquito species can significantly influence infection rates and mortality outcomes. Additionally, even if certain mosquitoes survive for extended periods, their fitness and reproductive capabilities may be compromised. Infected individuals may exhibit reduced lifespan and altered behaviors, which can limit their ability to spread malaria. Moreover, the application of Metarhizium in integrated pest management strategies can help target mosquito populations effectively, particularly when combined with other control measures, such as larvicides and environmental management. To clarify the phenomenon of survival, it is common for some mosquito species, especially those in the *Anopheles* genus, to exhibit variability in their susceptibility to fungal pathogens. Factors such as genetic diversity, environmental stressors. Further research on species-specific interactions and the ecological impact of Metarhizium can provide deeper insights into optimizing its use for malaria control. Even some infected mosquitoes survive more than 14 days, their ability to transmit malaria almost null at this time. We mentioned that in lines 146-148

 In this statement, “Indeed, laboratory studies showed that Metarhizium could be combined with chemical insecticides..” Please could you reference the laboratory studies and mention some of the key results from these studies.

**Response: **Thank you for your request for references and details regarding the laboratory studies on the combination of Metarhizium spp. with chemical insecticides

 Farenhorst *et al*., (2010) have explored this synergistic approach, demonstrating that combining Metarhizium with conventional insecticides can enhance the efficacy of pest control while potentially reducing the required dosage of chemical treatments. The study reported up to a 70% increase in mortality when the fungus was applied alongside the insecticide. We have added this in the text lines 141-143

 In this statement, “Combination of Metarhizium anisopliae with the fenitrothion insecticide was also shown to be effective against Blattella germanica..” This statement is confusing. I think you meant to say that combining M. anisopliae with fenitrothion increased the insecticide lethality (by what percentage??) in a cockroach.

**Response: **Thank you for pointing out the need for clarification regarding the effectiveness of combining Metarhizium anisopliae with fenitrothion against *Blattella germanica*. In the revised manuscript, we specified the increasement percentage.

 In the statement, “However, when the infection reach its lethal time (3 days post-infection),…” grammar, “reached” not “reach”. Also, is it lethal time or peak infection relating to increase in pathogen numbers?

**Response: **Thank you for your careful review and your suggestion regarding the terminology however "lethal time" is used correctly in the context of the manuscript. When mosquitoes come into contact with Metarhizium conidia, it typically takes about 3 days for the fungus to penetrate the mosquito cuticle and establish an infection. This period marks the beginning of the lethal time, during which the pathogen replicates and ultimately leads to the host's mortality. We corrected the grammatical error by changing "reach" to "reached" in the relevant statement.

 In this statement, “In this study, Farenhorst and colleagues evaluated the effect of fungal infection on insecticide efficacy. We think that for field orientated design, other option should assed..” this is a very vague statement, needs to be re-written.

**Response: **We revised the paragraph, it now reads: in that study, Farenhorst and colleagues evaluated the effect of fungal infection on insecticide efficacy.

 Furthermore, in the same statement, “…other option should assed” I think you meant assessed?

**Response: **Thank you, we correct "assessed" in the statement to ensure proper usage.

 In the same paragraph, I completely don’t understand what you mean. It seems you are saying that you are following up on some work that was done by others? However, you don’t give any references to whether it’s an ongoing work or published. Plus, is it from your group? hence the choice of text? 

**Response: ** No, this activity is not from our group. We reformat to let know that.

 In this statement, “…we tested whether locally isolated Metarhizium strains could…” Why locally?, does Metarhizium strains differ geographically? Brief explanation would helpful. **Response: ** Thank you for your question regarding the term "locally isolated" in reference to Metarhizium strains. “native strain” is more appropriate in this context.

 In this same paragraph, “In this work, we tested whether locally isolated Metarhizium strains could be used to increase insecticide tolerance in a highly resistant colony of Anopheles coluzzii mosquitoes in Burkina Faso..” Which is which? Increase or decrease insecticide tolerance. If you are using highly resistant colony, I assume that the goal is to decrease tolerance/resistance and that is what you are testing.

**Response: ** Thank you for your insightful observation regarding the phrasing in the manuscript. We appreciate your attention to detail. In the revised manuscript, we clarified that the objective of this work is to **decrease** insecticide tolerance in the highly resistant colony of *Anopheles coluzzii.*

 In statement, “First, we isolated a new Metarhizium pingshaense strain (S31) and tested its virulence...” you tested its virulence against what? Complete the statement even though it seems obvious. Thank you for your constructive feedback regarding the need for clarification in the statement.

**Response: ** We revised the manuscript to complete the sentence by specifying that we tested the virulence of the newly isolated Metarhizium pingshaense strain (S31) against *Anopheles coluzzii* mosquitoes.

 In statement, “We tested three sequential combinations, …” What do you mean by sequential? I am guessing you are referring to three different treatment groups. **Response: ** Thank you for your question regarding the term "sequential" in the statement. We appreciate your attention to clarity. We used sequential combinations to point out the specific order in which the treatments are applied

**General comments:**

- It is unclear to me how the main subject; *Metarhizium *contributes in terms of malaria control. Is it an adulticide or a synergist that increases potency of the insecticide? This did not come out clearly in the introduction. This cuts through with the rationale for the study which isn’t well articulated.
**Response: **  We revised the introduction to include the mode of action of *Metarhizium* on mosquitoes.
- The biology and application of *Metarhizium (*the main subject of the paper), isn’t properly discussed in the introduction.
**Response: ** We revised the introduction to include more details to *Metarhizium* biology and application

**Methodology**

**Specific comments:** In statement, “Three Metarhizium isolates, including two Metarhizium (Met) isolates (S10 and S26), were collected from Anopheles …” Probably better written as, “Three Metarhizium strains, including S10 and S26, were isolated from Anopheles …” replace “strains” not isolates and “isolated” not “collected”

**Response: **Thank You for proposing this rephrasing which we adopted in the text.

 Restructure your methodology; you collect mosquitoes first, then isolate the fungal strains **Response: **Thank you for your valuable feedback regarding the structure of the methodology section. We revised the manuscript to reflect the correct sequence of procedures.

 What was the rationale for collecting mosquitoes from house, woodpile and rhizosphere? Do they have different composition and distribution of Metarhizium?

**Response:**Thank you for your question. We collected mosquitoes from the house, woodpile and from rhizosphere. Rhizosphere is the part of plant roots in contact with soil. It has been shown that Metarhizium can be found there. Here we just mentioned places where we got Metarhizium. If not, we collected in other placed and insects other than mosquitoes. Regarding your question on composition and distribution of Metarhizium, different environments can have varying compositions and distributions of Metarhizium. Factors such as humidity, temperature, and the presence of organic matter can influence which strains thrive in each location. We specify that in lines 177-178.

  Maybe you could add a figure of the microscopy identification (in results section) since it is mentioned in the methodology.

**Response: **We don’t think that it is necessary to add pictures microscopy here as it can’t help make difference between our strains and others. 

 How did you know that the field mosquitoes were An. coluzzii? there is no mention of species genotyping. Do Metarhizium infection differ by mosquito species?

**Response: **Thank you for letting know your concern about the mosquito strain we used. We can ensure you that it was *An. coluzzii*. The mosquitoes we used in this experiment are lab strain. This strain called An. coluzzii VKPER is maintain in our lab at IRSS and was molecularly identify as *An. coluzzii*. We clarified in the text by adding the relative reference to this strain lines 165-166.

 How many mosquitoes did you collect and using which method?

**Response: **We used lab mosquitoes so we did not need a specific mosquito collection method. As mention in lines 203-204, we used 100 females An. coluzzii per fungi strain in 4 replicates. 

 What was the prevalence of Metarhizium in the wild mosquito population? (Show the results for this).

**Response: **Overall, 300 mosquitoes were collected from 3 types of resting sites (inhabited houses, abandoned houses and outdoor piles of wood). Two mosquitoes were positive to Metarhizium infection as previously reported by Bilgo et al, (2018). We didn’t report this result as it is not from this current study even if it is from our group. We briefly reported the method in lines 224-226.

   How did you isolate the Metarhizium strains and from which mosquito organ? Did you crash them or put a whole mosquito? Please clarify and how many mosquitoes did you have to use to isolate the fungi?

**Response:**Thank you for these questions. Metarhizium strains were isolated from whole mosquitoes on fungal selective medium contained 42 g potato-dextrose agar, 0.5 g chloramphenicol and 0.6 g cetyl trimethylammonium bromide per litter (Bilgo et al, 2018). One mosquito is enough to isolate Metarhizium if it is infected. We specified that in the method section, lines 178-181.

 The subheading, “Fungal determination formulations for bioassays.” Is very confusing. Are you trying to mean serial dilutions?

**Response: **Thank you for your insightful observation regarding the phrasing. The correct phrasing is: Determination of fungal formulation concentration for bioassays.

  You used homogenisation, and I’m wondering homogenisation doesn’t destroy the cells? Perhaps not since fungi are tougher but If you could please briefly state the reason for some of these steps for the audience to understand and follow.

**Response: **Thank you for your rising this concern about homogenizing the fungal solution. We have checked that homogenisation does not destroy or affects conidia viability. We have also added more rationale to these steps in the text for better understanding.

   In the line, “An improved counting method was used to determine the stock solution ….” Maybe you should briefly state the basis of this improved counting method.

**Response: ** We added the details on the fungal solution concentration determination.

  In the line, “Valley du Kou in Burkina Faso and then selected repeatedly to fix the kdr gene…”  I don’t think that the selection was fix the kdr but rather maintain resistance of the colony but in the process, kdr gets fixed because it is a strong resistance maker

**Response: **Thank you for clarifying the nuance between fixing and maintaining a marker. We took it into account.

 This subheading bioassays, do you mean fungal bioassays? which I think you could rather use fungal infection experiments OR insecticide bioassays. Please clarify. Also, why did you use spraying and equipment did you use for this? Couldn’t you deliver the fungi through sugar feeding? 

**Response: **Mosquitoes were infected by spraying them with the fungal solution using an atomizer. *Metarhizium* infects mosquitoes through direct contact with their cuticle **Goettel, M. S., & Inglis, G. D. (1997)**. We added that to the manuscript.

 In the line, “…..solution for the controls before their reanimation.” “reanimation” is the wrong word I think. better to use “recovery”

**Response: **Thank you for your suggestion. We agree that 'recovery' is a more appropriate term in this context, as it accurately reflects the process intended. We have revised the manuscript accordingly."

 Why were dead mosquitoes collected twice a day? And at what times/periods and why? **Response:**The rationale behind collecting the mosquitoes twice a day in the morning and in the evening was to prevent them drying out in the cage. When the mosquito dries out it crumbles when you try to remove it and this can also reduce the growth of Metarhizium on the mosquito corpses.

  Why were dead mosquitoes washed? at least briefly state the rationale for this. **Response: **The reason behind washing mosquitoes before culturing them is to eliminate any other contaminants that might be present on the mosquito cuticle or even Metarhizium conidia would not have been able to penetrate the mosquito cuticle and infect it. This makes it possible to identify mosquitoes that have actually been infected by Metarhizium to those that dead for other reasons. 

 In the subheading, “Natives Metarhizium pingshaense and deltamethrin compatibility test.” I think you mean strains?

**Response: **Thank you for pointing that out. You are correct; 'strains' is more precise in this context. We have revised the subheading to 'Strains of *Metarhizium pingshaense* and deltamethrin compatibility test.

  I can see the results for deltamethrin-only treatment group but can’t see the description of how the group was obtained in the methodology section.

**Response: **Thanks for pointing out this inconsistency in the methodology. That group was obtained from mosquitoes expose to deltamethrin alone. We clarified it the methodology.

**Results**

**Specific comments:** If possible and applicable, you should include the results showing the prevalence of fungal strains in field mosquitoes.

**Response: **Thank you for this valuable suggestion. Unfortunately, we did not collect prevalence data of the fungal strains in field mosquito populations for this study. However, we agree that such information would provide additional context on the natural occurrence of these strains.

   In subheading, “Native strains of Metarhizium pingshaense virulence against insecticide-resistance mosquito line Anopheles coluzzii”.I think you mean “insecticide-resistant…” **Response: **Thank you for noticing this. We agree with your suggestion and will change 'insecticide-resistance mosquito line' to 'insecticide-resistant mosquito line' to improve clarity."

  Why did you get such high mortality when exposed to control solution?

**Response:**The high mortality in the control mosquitoes is due to the natural mortality of the mosquitoes as the mortality follow-up lasted 14 days.

   I’m failing to understand your figure 1. Were mosquitoes exposed to fungi without deltamethrin. If so, then what is the use of adding deltamethrin if the fungi alone can offer significant mortality? please clarify and also add some p-values in the graph comparing the different groups.

**Response:**It is true that fungi alone can achieve significant mortality. As chemical insecticide is wildly used in malaria endemic countries for mosquitoes control, the purpose of this study was to see if *Metarhizium* and chemical insecticide can be used conjointly to achieve higher mortality. We will add p value to the figure

 Instead of, “Survival of mosquitoes following the three types of combination sequences [M. pingshaense and Deltamethrin 0.05%]” , You could write; “Survival of mosquitoes following exposure to M. pingshaense and Deltamethrin” 

**Response:**Thank you for this suggestion. We agree that 'Survival of mosquitoes following exposure to *M. pingshaense* and Deltamethrin' provides a clearer description. We revised the subheading accordingly

  I think instead of using combination sequence, rather use exposure groups

**Response: **Thank you for your suggestion. We agree that exposure groups are a more precise term than combination sequence. We have changed this throughout the manuscript to enhance clarity.

   In the paragraph, “We then tested if these locally isolated fungal strains could increase susceptibility to insecticides in a highly resistant strain of Anopheles coluzzii. We used three approaches: 1) simultaneous, where mosquitoes were exposed to a standard dose of deltamethrin and 107 conidia/ml dose of fungi at the same time; 2) fungi after insecticide, where the same fungal dose was applied to mosquitoes 24 hours after exposure to standard deltam- ethrin; 3) fungi before insecticide, where mosquitoes were exposed to standard deltamethrin three days after being infected with the same fungal dose.” This is a repetition. you already mentioned this in the method section. Probably delete; “….We used three approaches: 1) simultaneous, where mosquitoes were exposed to a standard dose of deltamethrin and 107 conidia/ml dose of fungi at the same time; 2) fungi after insecticide, where the same fungal dose was applied to mosquitoes 24 hours after exposure to standard deltam- ethrin; 3) fungi before insecticide, where mosquitoes were exposed to standard deltamethrin three days after being infected with the same fungal dose.”

**Response: **Thanks for pointing out this repetition, we revised the manuscript removing the repeated section.

   You should have characterized the resistance profile of the mosquitoes (field and colony) or mention it from previous studies.

**Response: **Thank for your suggestion, we used wild mosquitoes for fungi isolation, but not for the bioassays where we used a laboratory insecticide resistant *An. coluzzii* colony. This colony is well characterised and known to be highly resistant to deltamethrin the insecticide we used. This was mentioned in the text lines 165-167.

   In the statement, “…..suggesting that the insecticide can have a deleterious effect” I think antagonistic sounds better than deleterious and should replace everywhere.

**Response: **Thank you for pointing this out. We agree that 'antagonistic' more accurately describes the effect in this context. We will replace 'deleterious' with 'antagonistic' throughout the manuscript to improve clarity and precision.

 Why did you use the Cox proportional hazard analysis instead of the Kaplan-Meier survival analysis? Please briefly state the rationale.

**Response: **We opted for the Cox proportional hazards model instead of the Kaplan-Meier survival analysis because the Cox model allows for the inclusion of covariates, which was essential for our study; while Kaplan Meier survival analysis does not allow. This approach enabled us to assess the influence of covariates including treatment, mosquito strain and even their interaction on survival.

   Why is there no control and deltamethrin-alone in figures B, C and D? Please include them for comparison.

**Response: **Thank you for highlighting this point. The control and deltamethrin-alone groups are indeed the same across all panels, and we initially omitted them in Figures B, C, and D to avoid repetition. However, we agree that including them in each figure would facilitate clearer comparisons. We added these groups to enhance the comparative analysis.

   Please revise the Figure 2 legend. It is not succinct. You don’t need to mention “3 to 5 day old mosquitoes” everywhere. that’s not the best description of that figure. please refer to your sentences; “Specifically, when Metarhizium was applied on its own, it increased the risk of death from 6.7 to 7.5 fold depending on the strain; in contrast, deltamethrin effect was similar to control (Hazard ratio = 1.2), as expected in a highly insecticide resistant strain (Figure 2A).” **Response: ** Thank you for your feedback. We understand the importance of making the Figure 2 legend more concise and focused. We revised the legend to remove repetitive details, it now reads: *Effect of Metarhizium and deltamethrin on survival in insecticide-resistant Anopheles mosquitoes. Metarhizium application alone (A). Metarhizium application before deltamethrin (B). Metarhizium application after deltamethrin (C). Co-formulation of Metarhizium and deltamethrin (D).*

**Discussion**

**Specific comments:** Instead of’ “In this study we tested if locally isolated strains of Metarhizium pingshaense entomopathogenic fungi ..” you could write; “In this study we tested if locally isolated strains of an entomopathogenic fungi, Metarhizium pingshaense …” **Response: ** Thank you for this suggestion. We revised this sentence accordingly.

 In the line, “….were applied before permethrin in a field population…” How did permethrin come into the picture here? I’m guessing you are trying to discuss a similar study. If so, please revise your sentence construction to make it clearer.

**Response: ** You are correct; we were referring to findings from a similar study involving permethrin to provide context. We revised the sentence to clarify that this example is from previous research.

   “fungal metabolites?” could you please expound on this more?

**Response:**Thank you for this request for clarification. We have expanded on the term 'fungal metabolites' to provide additional context. Specifically, we refer to the bioactive compounds produced by *Metarhizium pingshaense*, which can contribute to the pathogenicity of the fungus in mosquitoes. A well known of these bioactive compounds is destruxin. We have integrated this insight to further contextualize the potential mechanisms at play in our study. The revision now reads: fungal bioactive compounds like destruxins.

 In the statement, “Another mechanism might rely on disruption of cuticular integrity by the fungus….” this statement is attached to what point? I am guessing you are trying to discuss the fungal mechanisms contributing to mosquito susceptibility. You need to introduce the subject on fungal mechanisms properly.

**Response:**You are correct; the statement refers to fungal mechanisms that may contribute to increased mosquito susceptibility to deltamethrin. We revised the introduction of this section to clearly introduce fungal mechanisms, including cuticular disruption, as factors that enhance insecticide susceptibility.

   You mention, “Another mechanism might rely on disruption of cuticular integrity by the fungus” …what is your reference and experimental evidence for this if there are any?

**Response:**The suggestion that *Metarhizium* fungi might disrupt cuticular integrity is based on previous research showing that entomopathogenic fungi can erode the insect cuticle to facilitate host infection (Ortiz-Urquiza and Keyhani, 2013). However, in our study, we did not directly assess cuticular disruption. We included this hypothesis as a potential explanation for the observed increased susceptibility to insecticides. We have provided appropriate references to support the proposed mechanism.

 In the line, “…making mosquitoes more susceptible to insecticides and leading to swift mortality” what is swift mortality. The word seems redundant and you could remove. We agree that 'swift' may be redundant in this context.

**Response:**We revised the sentence to simply state 'making mosquitoes more susceptible to insecticides and leading to mortality’

   In the paragraph, “The survival of mosquitoes infected with Metarhizium pingshaense three days after deltamethrin exposure was similar to the survival of Metarhizium pingshaense applied alone.” Could you please test statistically? Also, again, if Metarhizium alone can work, what is the use of combining it with deltamethrin? please discuss

**Response:**While *Metarhizium* alone may lead to mortality, in our study we tested if in combination with deltamethrin it could enhance its effectiveness, by decreasing insecticide tolerance in resistant populations. We showed that if fungi are applied before deltamethrin, risk of death (Hazard Ratio, HR = 13.7-15.43 depending on strain) is significantly increased compared to deltamethrin (HR = 1.21) alone or control (HR = 1). If applied after deltamethrin (HR = 6.62-7.45 depending on strain), there is no significant difference than when applied alone (HR = 6.68-7.53 depending on strain) – most likely as the insecticide is not present in the mosquito by the time the fungi become virulent. More details on the statistics are presented in the text and in Table 1. We have expanded our discussion to clarify these points.

   I think the discussion on the virulence results should come first.

**Response:**By discussing the combination result first we want to highlight the most important result of our study first.

   In the statement, “The similarity between the mortalities of the three tested isolates could be due to their geographical proximity ..” Is it possible to perform a phylogenetic tree of relatedness between your isolates and other known isolates from other regions to back up your claim?

**Response: **Thank you for this suggestion. While we did not perform sequencing in this study, we acknowledge that constructing a phylogenetic tree of relatedness could strengthen our claim regarding the similarity between the tested strain due to geographical proximity. Unfortunately, without sequencing data, we are unable to conduct this analysis at this time, but we will consider for follow up studies.

   In the statement, “At the intermediate concentration of 10 ^7^ conidia/ml, no isolate reached the 80% mortality threshold..” I think the 80% threshold is for insecticides. Does this also apply for biological agents?

**Response: **You are correct that the 80% mortality threshold is often used in the context of insecticides. While this threshold is commonly referenced for assessing the effectiveness of chemical insecticides, it may not directly apply to biological agents like *Metarhizium pingshaense*. We have revised the statement to specify that this threshold is typically associated with insecticide efficacy.

   In the statements, “…..the same concentration25. However, a study conducted in Benin by Howard et al. reported similar results42.” if the study reported similar results, why do you say “however…” please revise the sentence construction.

**Response: **We agree that it is misleading since both studies reported similar results. We have revised the sentence to replace 'however' with a more appropriate transition, such as 'additionally,' to accurately reflect the supportive nature of the findings.

   You discuss here, “This finding could lead to a new IPM strategy for malaria vector control using Metarhizium pingshaense as an IRS product in conjunction with the use of LLINs. The use of Metarhizium-impregnated fabrics placed in strategic mosquito resting places could also be considered while maintaining the current vector control strategies” Based on your results, don’t you think this strategy would fail? Since the mosquitoes would be exposed to both insecticide and fungi concurrently ..perhaps application in larval stages would work? or maybe apply Metarhizium alone? Clarify the discussion on how you would apply this.

**Response: **As long as *Metarhizium* and chemical insecticide are not applied on the same fabric or surface, the strategy will work. The strategy will fail as soon as fungi conidia comes directly in contact with the chemical insecticide. Based on this evidence, we believe that the strategy will not fail.

 You discuss here, “Metarhizium pingshaense could be used as an alternative to replace chemical insecticides;”. This is not correct. The goal of IVM isn’t to replace what works but complement existing methods.

**Response: **  Thank you for this important clarification regarding integrated vector management (IVM). We appreciate your insight that the goal of IVM is not to replace effective chemical insecticides but to complement them with biological alternatives. In a context of insecticide resistance in mosquitoes new tools and strategies are very needed. Here we wanted to point out that Metarhizium can kill mosquitoes when applied its own but when it’s applied as comment to chemical insecticide, we achieve better result.

 You mention here, “….however, their combination may offer strong synergy..” I don’t think it would be feasible to combine them since you would have to find a way to infect mosquitoes first for days before exposing them to insecticides. Please discuss more.

**Response: ** We understand your concern about the feasibility of this combination. The idea here is to apply the Metarhizium to the mosquitoes' resting surfaces so that they will be infected there and then when they go in search of a blood meal they will be exposed to the insecticide through the LLINs. In general, the approach aims at increasing the chance that an insecticide resistance mosquito will become susceptible by the infection of the fungi. While not all mosquitoes will be in contact with the insecticide after infection, on a population level, this is expected to increase the efficacy of insecticides overall.

   You mention here, “…..improving Metarhizium virulence against insecticide-resistant mosquitoes.” I thought Metarhizium improves insecticide lethality. Clarify! Indeed, Metarhizium improves the effectiveness of deltamethrin. However, this study and previous works, showed that different strains possess different virulence. Here we want to point out that it improving the performance of Metarhizium could be achieved to make it a more effective tool.

  In the statement, “However, it is necessary to better understand the synergy observe in ..” there is a grammatical error here (observed nor observe).

**Response: **Thank you for your careful attention to the grammatical error.

**General comments:** If *Metarhizium *occurs naturally in mosquito species, then why isn’t this synergistic effect observed in wild resistant mosquito populations? The discussion needs to come out clearly.

**Response: ** Thank you for this important observation. We can ensure that mosquitoes strain we used for bioassays in this study are all laboratory strain and Metarhizium free. Soo the synergistic effect we observed cannot be from natural infection. Also, while these fungi are locally adapted, they rarely infect mosquito populations at very high prevalence (mention some study /data if you have). So, we first need to infect them at high prevalence to obtain an effect.

 Parts of the discussion need to be re-written. Some of the paragraphs are confusing to read and there are no connecting words or sentences. It is hard to follow. Furthermore, the discussion is a bit shallow and could use more explanations.

**Response: ** Thank you for your valuable feedback. We have revised the discussion section in line with your suggestions, adding connecting phrases and transitions to improve readability .and coherence. Additionally, we expanded on key points to provide a more in-depth interpretation of our findings, ensuring a clearer and more comprehensive discussion.

**Reviewer 3** Microbes have conventionally been used or proposed to control disease vector populations, as an alternative to insecticides.  However, delayed mortality, variation in production batches, loss of virulence in the natural environment, and cost have influenced the uptake of these microbes. This study adds to the growing evidence that microbes have sub-lethal effects that can also contribute to disease control in addition to the reduction of vector populations. Understanding the sub-lethal effects can inform how microbes can be used to complement core disease control tools. Please see my comments below. Note that this is in addition to general comments from the first reviewer. I support the comment that there is a need to improve the writing to make it clear and concise and ensure there are no spelling and grammatical mistakes.

**Major comments:** The authors report a new strain (S31). However, no data or analysis (sequencing, ANI comparison) is provided to support this claim.

**Response: ** Thank you for highlighting this point. We recognize that data such as sequencing or Average Nucleotide Identity (ANI) comparisons would provide robust support for confirming the uniqueness of strain S31. While, we did not conduct genomic sequencing for this strain in the current study, Met_S31 was confirmed as *Metarhizium pingshaense* through Polymerase Chain Reaction (PCR) of the Internal Transcribed Spacer (ITS). Additionally, we characterized it based on macro-morphological characters, such as conidiogenesis, estimation of radial growth, spore color and mycelia texture of the isolates on PDA media according to Humber (Humber RA, 2005). In addition, we used microscopic morphology to identify Metarhizium spp. spores as described by Fernandes et al. (2010). We have now clarified this in the manuscript and refer to Met_S31 isolate instead of strain to avoid confusion.

   The mosquitoes were exposed to fungus and the insecticide in three sequences. In the sequence where mosquitoes are simultaneously exposed to insecticide and fungus, the fungus spores are sprayed on the insecticide-treated surface and the mosquitoes are exposed to the surface. While in the fungi-only or fungi-first treatments, the mosquitoes are sprayed directly. This methodology does not ensure that mosquitoes acquire the same number of spores; even if the spores’ concentration was similar, mosquitoes probably picked fewer spores in the simultaneous exposure. The comparison therefore is not justified. Spraying mosquitoes directly does not reflect what will happen in natural settings. In a natural setting, mosquitoes will most likely pick the spores by landing/rubbing against treated surfaces, hence fewer spores will be picked.

**Minor comments:** Abstract: Results: Remove “Therefore”.

**Response: ** Thank you for this suggestion. We removed 'Therefore' from the Results section.

   Abstract: Conclusion: Fungus name needs to be in Italics.

**Response: ** Thank you for pointing that out.

 Heading Plain language summary: “Plaine” not “plan”.

**Response: ** Thank you we addressed that

   Plain language summary: Please rephrase “Mosquito control products made from a type”; the products are fungus spores and so the fungus itself, so the statement “made from fungus” is not correct.  

**Response: **Thank you for this observation. We agree that the phrase could be clearer. We will rephrase it to specify that the mosquito control products consist of fungal spores, rather than being 'made from fungus.' For example, we could revise it to 'Mosquito control products that use fungal spores as active ingredients.'

 Background: first paragraph: “ we assisted in a stable….”. what does “we” mean? Thank you for catching this ambiguity.

**Response:**We revised the manuscript to remove the confusion.

   Background: second paragraph: “Although are useful in some population, but might be less effective in other populations that are very strongly resistant”. Is this a human or mosquito population?

**Response:**Thank you for pointing out the need for clarity here. The statement refers specifically to mosquito populations. We revised the sentence to clearly indicate that some mosquito populations may respond differently, with certain highly resistant mosquito populations showing reduced effectiveness to these interventions.

 Background: second paragraph: “….address insecticide resistance in malaria vector control still crucial.”  Add “is” before “still crucial.”

**Response: **Thank you for catching this. We will add 'is' before 'still crucial' to correct the sentence structure.

 Background: third paragraph: Integrated Vector Management is the term generally used. The reference 18 does not support the statement. The statement is general and there are better references for this.

**Response: **Thank you for this helpful feedback. We will revise the terminology to 'Integrated Vector Management' for consistency with standard usage. Additionally, we have supplemented reference 18 with more relevant citations that better support the general statement on Integrated Vector Management.

 Background: sixth paragraph: Remove “In this study”

**Response: **Thank you for your suggestion. We have removed the phrase 'In this study' from the sixth paragraph.

   Methods: The terms “strains”, “colony”, “line” and “isolate” are used interchangeably both for mosquitoes and fungus. Please choose one with justification and be consistent.

**Response: **Thank you for pointing out the inconsistency in terminology. We have selected a specific term for each context: 'strain' is now used to refer to the fungus, and 'colony' is used for the mosquito populations.

   Methods: Bioassay: “….14 days to determine the transmission potential of mosquitoes after *Metarhizium*…”. The transmission potential was not determined. Rephrase.

**Response: **Thank you for bringing this to our attention. We rephrased the sentence to accurately reflect the intent of the bioassay.

   Result section: Please remove the methodology from the result sections to avoid repetition.

**Response: **Thank you for your feedback. We removed any methodological descriptions from the Results section to eliminate repetition and ensure that this section focuses solely on the presentation of our findings.

**Data analysis: Why use two methods: GLM and survival analysis? Also for cox regression model would be more insightful if the fungus strain is used as a factor**. **Response: **Thank you for your insightful question. We employed GLM for fungi strain virulence against An. coluzzii as we considered total mortalities after 14 days Metarhizium infection. Four combinations efficacy, we used survival analysis as mortalities were monitored every day up to 14 days.

 Fungus strain was included as a factor in both models. In the methods we stated: “ Mortality of mosquitoes 14 days after exposure to fungi was analysed using generalized linear models using binomial distribution, fungal strain (control, S10, S26, and S31) as fixed effect and replicates as random effect. Survival over several days after treatment was analysed using cox proportion hazard models using treatment (control, deltamethrin, S10, S26, and S31) and sequence (alone, fungi before deltamethrin, fungi after deltamethrin, fungi with deltamethrin) as fixed effects, and replicates as random effect.”

 Please report the 95% CI for the Hazard ratios. Also, can HR values be interpreted as “synergism”. There are methods to calculate synergism including one used in the reference cited in the manuscript (Farenhorst, 2010).

**Response: **Thank you for your valuable suggestions. We have reported the 95% confidence intervals (CI) for the Hazard Ratios (HR) in the results to provide a clearer understanding of the precision of our estimates. Synergy is when combined effect of multiple treatments give greater effect than one each treatment. Here we estimate it throughout comparison of HR of combination of fungi and deltamethrin and HR of each one alone. While there are others to calculate synergism, HR can also be used to estimate it.

 Figure 2 separates what needs to be compared. My suggestion is to separate the graphs based on the fungus strains and each graph allows the comparison of fungus only, fungus first, Deltamethrin first, and simultaneous exposure.

**Response: **Thank you for your suggestion of separating the figure2 based on the fungus strains. However, by separating based on the type of treatment, we want to highlight the most virulent combination. Also, there is no too much difference between fungi strains.

   Conclusion: “ *Metarhizium pingshaense* could be used as an alternative to replace chemical insecticides…” This study does not support the use of fungus to replace chemical insecticides. **Response: ** Thank you for your comments. We revised the text to clarify that while *Metarhizium pingshaense* is not proposed as a direct alternative to replace chemical insecticides, its combination with deltamethrin could enhance the virulence of the fungus against insecticide-resistant mosquitoes. We emphasized that this combination is aligned with the Integrated Vector Management approach, which is strongly recommended by the WHO for managing insecticide resistance in malaria control efforts.

# Reviewer report: Reviewer response for version 1

**DOI:** 10.21956/wellcomeopenres.23487.r93094

**Published:** 2024-09-06

**Authors:** Ambrose Oruni

**Affiliations:** 1 Liverpool School of Tropical Medicine, Liverpool, UK

**Overall comments:**

- This manuscript has the potential to be a very good piece of work. However, the authors need to carefully address the comments below and add additional work if possible. The science is there.
- The paper requires a major revision of the writing style. A lot of the sentences including figure legends require reconstruction to correct the grammar, spelling mistakes, flow and the use of the right words and also remove redundancy.

**Introduction:**

**Specific comments:**

- Wouldn’t it be better to introduce the disease first and vector/malaria control?
- I don’t think the language used here i.e. “we assisted …” is appropriate.
- Reference 2 is WHO malaria report 2015, please update to the current.
- I think your references are misplaced, Bhatt et al talked about “averted malaria cases” not “reduction” … The 60% paragraph should have Bhatt et al reference not WHO…please revise.
- You mention that “…. early 2000 have led to impressive advances in malaria control….” What are these advances? Briefly state them.
- In this statement; “The spread of insecticide resistance in malaria vectors has led to a drop in the effectiveness of these tools, resulting in a resurgence of the disease since 2015” …this is not entirely correct. Insecticide resistance is part but there could be other factors. Please revise
- In this statement, “…with 19 billion ITNs supplied to the region from 2004 to 2019…” It should be “approximately or over 19 billion”
- In this statement, “…. development of strategies to avert the selection of resistance..” I think using “strategies to reverse insecticide resistance…” sounds better because it’s probably impossible to avert it now..
- In this statement; “Although are useful in some population, but might be less effective in other populations that are very strongly resistant ..” you could replace with; “Although PBO nets are still effective in some mosquito populations, they show significant reduction in efficacy in other populations that are highly resistant..” with references
- In this statement, “…develop new class of insecticide limit the practical options for simple chemical-based insecticide resistance management..” Why simple? Plus, i expected you to reference more studies reporting cross resistance here. I thought the reference on Fungal entomopathogens should come in later.
- In this statement, “One solution has been identified in the integrated vector control (IVC), which is a combination of different tools and strategies for controlling a single vector”.  IVC combines different malaria control strategies, so your statement is confusing. Please state the one solution you are referring to, otherwise, revise the statement.
-  In this statement, “Entomopathogenic fungi are probably the most common natural insect pathogens…” Are you sure they are? If so, then support the statement with some studies and evidence. Also briefly mention what other pathogens are there.
- In this statement, “…development as they do not need to be ingested, in contrast to bacteria and viruses (put full stop here). Indeed….”
- Plus, how do the fungi occur in nature? How do they infect the mosquitoes, is it vertically, horizontally or both? What is the rate of spread in population once infected? All this information is missing
- Could you please give more information about the biology of this Metarhizium spp?
- In this statement, “Specifically, Metarhizium requires at least 3 days to infect mosquitoes, mortalities occur within 7 days, and certain mosquitoes survive even 14 days post-infection…” If it is less effective and act slower with mosquitoes surviving even up to 14 days inoculation period, how is this a good control method for malaria?
  Maybe you could explain these scenarios where mosquitoes survive longer and why? Is it a common phenomenon that most mosquitoes survive longer and which ones do?
- In this statement, “Indeed, laboratory studies showed that Metarhizium could be combined with chemical insecticides..” Please could you reference the laboratory studies and mention some of the key results from these studies.
- In this statement, “Combination of Metarhizium anisopliae with the fenitrothion insecticide was also shown to be effective against Blattella germanica..” This statement is confusing. I think you meant to say that combining M. anisopliae with fenitrothion increased the insecticide lethality (by what percentage??) in a cockroach.
- In the statement, “However, when the infection reach its lethal time (3 days post-infection),…” grammar, “reached” not “reach”. Also, is it lethal time or peak infection relating to increase in pathogen numbers?
- In this statement, “In this study, Farenhorst and colleagues evaluated the effect of fungal infection on insecticide efficacy. We think that for field orientated design, other option should assed..” this is a very vague statement, needs to be re-written.
- Furthermore, in the same statement, “…other option should assed” I think you meant assessed?
- In the same paragraph, I completely don’t understand what you mean. It seems you are saying that you are following up on some work that was done by others? However, you don’t give any references to whether it’s an ongoing work or published. Plus, is it from your group? hence the choice of text?
- In this statement, “…we tested whether locally isolated Metarhizium strains could…” Why locally?, does Metarhizium strains differ geographically? Brief explanation would helpful.
- In this same paragraph, “In this work, we tested whether locally isolated Metarhizium strains could be used to increase insecticide tolerance in a highly resistant colony of Anopheles coluzzii mosquitoes in Burkina Faso..” Which is which? Increase or decrease insecticide tolerance. If you are using highly resistant colony, I assume that the goal is to decrease tolerance/resistance and that is what you are testing.
- In statement, “First, we isolated a new Metarhizium pingshaense strain (S31) and tested its virulence...” you tested its virulence against what? Complete the statement even though it seems obvious.
- In statement, “We tested three sequential combinations, …” What do you mean by sequential? I am guessing you are referring to three different treatment groups.

**General comments:**

- It is unclear to me how the main subject; *Metarhizium *contributes in terms of malaria control. Is it an adulticide or a synergist that increases potency of the insecticide? This did not come out clearly in the introduction. This cuts through with the rationale for the study which isn’t well articulated.
- The biology and application of *Metarhizium (*the main subject of the paper), isn’t properly discussed in the introduction.

**Methodology**

**Specific comments:**

- In statement, “Three Metarhizium isolates, including two Metarhizium (Met) isolates (S10 and S26), were collected from Anopheles …” Probably better written as, “Three Metarhizium strains, including S10 and S26, were isolated from Anopheles …” replace “strains” not isolates and “isolated” not “collected”
- Restructure your methodology; you collect mosquitoes first, then isolate the fungal strains
- What was the rationale for collecting mosquitoes from house, woodpile and rhizosphere? Do they have different composition and distribution of Metarhizium?
- Maybe you could add a figure of the microscopy identification (in results section) since it is mentioned in the methodology
- How did you know that the field mosquitoes were An. coluzzi? there is no mention of species genotyping. Do Metarhizium infection differ by mosquito species?
- How many mosquitoes did you collect and using which method?
- What was the prevalence of Metarhizium in the wild mosquito population? (Show the results for this).
- How did you isolate the Metarhizium strains and from which mosquito organ? Did you crash them or put a whole mosquito? Please clarify and how many mosquitoes did you have to use to isolate the fungi?
- The subheading, “Fungal determination formulations for bioassays.” Is very confusing. Are you trying to mean serial dilutions?
- You used homogenisation, and I’m wondering homogenisation doesn’t destroy the cells? Perhaps not since fungi are tougher but If you could please briefly state the reason for some of these steps for the audience to understand and follow.
- In the line, “An improved counting method was used to determine the stock solution ….” Maybe you should briefly state the basis of this improved counting method.
- In the line, “Valley du Kou in Burkina Faso and then selected repeatedly to fix the kdr gene…”  I don’t think that the selection was fix the kdr but rather maintain resistance of the colony but in the process, kdr gets fixed because it is a strong resistance maker
- This subheading bioassays, do you mean fungal bioassays? which I think you could rather use fungal infection experiments OR insecticide bioassays. Please clarify. Also, why did you use spraying and equipment did you use for this? Couldn’t you deliver the fungi through sugar feeding?
- In the line, “…..solution for the controls before their reanimation.” “reanimation” is the wrong word I think. better to use “recovery”
- Why were dead mosquitoes collected twice a day? And at what times/periods and why?
- Why were dead mosquitoes washed? at least briefly state the rationale for this.
- In the subheading, “Natives Metarhizium pingshaense and deltamethrin compatibility test.” I think you mean strains?
- I can see the results for deltamethrin-only treatment group but can’t see the description of how the group was obtained in the methodology section.

**General comments:**

- The methodology of the paper seems to be missing some key steps as mentioned above.

**Results**

**Specific comments:**

- If possible and applicable, you should include the results showing the prevalence of fungal strains in field mosquitoes.
- In subheading, “Native strains of Metarhizium pingshaense virulence against insecticide-resistance mosquito line Anopheles coluzzii”  ..I think you mean “insecticide-resistant…”
- Why did you get such high mortality when exposed to control solution?
- I’m failing to understand your figure 1. Were mosquitoes exposed to fungi without deltamethrin. If so, then what is the use of adding deltamethrin if the fungi alone can offer significant mortality? please clarify and also add some p-values in the graph comparing the different groups.
- Instead of, “Survival of mosquitoes following the three types of combination sequences [M. pingshaense and Deltamethrin 0.05%]” , You could write; “Survival of mosquitoes following exposure to M. pingshaense and Deltamethrin” 
- I think instead of using combination sequence, rather use exposure groups
- In the paragraph, “We then tested if these locally isolated fungal strains could increase susceptibility to insecticides in a highly resistant strain of Anopheles coluzzii. We used three approaches: 1) simultaneous, where mosquitoes were exposed to a standard dose of deltamethrin and 107 conidia/ml dose of fungi at the same time; 2) fungi after insecticide, where the same fungal dose was applied to mosquitoes 24 hours after exposure to standard deltam- ethrin; 3) fungi before insecticide, where mosquitoes were exposed to standard deltamethrin three days after being infected with the same fungal dose.” This is a repetition. you already mentioned this in the method section. Probably delete; “….We used three approaches: 1) simultaneous, where mosquitoes were exposed to a standard dose of deltamethrin and 107 conidia/ml dose of fungi at the same time; 2) fungi after insecticide, where the same fungal dose was applied to mosquitoes 24 hours after exposure to standard deltam- ethrin; 3) fungi before insecticide, where mosquitoes were exposed to standard deltamethrin three days after being infected with the same fungal dose.”
- You should have characterized the resistance profile of the mosquitoes (field and colony) or mention it from previous studies.
- In the statement, “…..suggesting that the insecticide can have a deleterious effect” I think antagonistic sounds better than deleterious and should replace everywhere.
- Why did you use the Cox proportional hazard analysis instead of the Kaplan-Meier survival analysis? Please briefly state the rationale.
- Why is there no control and deltamethrin-alone in figures B, C and D? Please include them for comparison.
- Please revise the Figure 2 legend. It is not succinct. You don’t need to mention “3 to 5 day old mosquitoes” everywhere. that’s not the best description of that figure. please refer to your sentences; “Specifically, when Metarhizium was applied on its own, it increased the risk of death from 6.7 to 7.5 fold depending on the strain; in contrast, deltamethrin effect was similar to control (Hazard ratio = 1.2), as expected in a highly insecticide resistant strain (Figure 2A).”

**Discussion**

**Specific comments:**

- Instead of’ “In this study we tested if locally isolated strains of Metarhizium pingshaense entomopathogenic fungi ..” you could write; “In this study we tested if locally isolated strains of an entomopathogenic fungi, Metarhizium pingshaense …”
- In the line, “….were applied before permethrin in a field population…” How did permethrin come into the picture here? I’m guessing you are trying to discuss a similar study. If so, please revise your sentence construction to make it clearer.
- “fungal metabolites?” could you please expound on this more?
- In the statement, “Another mechanism might rely on disruption of cuticular integrity by the fungus….” this statement is attached to what point? I am guessing you are trying to discuss the fungal mechanisms contributing to mosquito susceptibility. You need to introduce the subject on fungal mechanisms properly.
- You mention, “Another mechanism might rely on disruption of cuticular integrity by the fungus” …what is your reference and experimental evidence for this if there are any?
- In the line, “…making mosquitoes more susceptible to insecticides and leading to swift mortality” what is swift mortality. The word seems redundant and you could remove.
- In the paragraph, “The survival of mosquitoes infected with Metarhizium pingshaense three days after deltamethrin exposure was similar to the survival of Metarhizium pingshaense applied alone.” Could you please test statistically? Also, again, if Metarhizium alone can work, what is the use of combining it with deltamethrin? please discuss
- I think the discussion on the virulence results should come first.
- In the statement, “The similarity between the mortalities of the three tested isolates could be due to their geographical proximity ..” Is it possible to perform a phylogenetic tree of relatedness between your isolates and other known isolates from other regions to back up your claim?
- In the statement, “At the intermediate concentration of 107 conidia/ml, no isolate reached the 80% mortality threshold..” I think the 80% threshold is for insecticides. Does this also apply for biological agents?
- In the statements, “…..the same concentration25. However, a study conducted in Benin by Howard et al. reported similar results42.” if the study reported similar results, why do you say “however…” please revise the sentence construction.
- You discuss here, “This finding could lead to a new IPM strategy for malaria vector control using Metarhizium pingshaense as an IRS product in conjunction with the use of LLINs. The use of Metarhizium-impregnated fabrics placed in strategic mosquito resting places could also be considered while maintaining the current vector control strategies” Based on your results, don’t you think this strategy would fail? Since the mosquitoes would be exposed to both insecticide and fungi concurrently ..perhaps application in larval stages would work? or maybe apply Metarhizium alone? Clarify the discussion on how you would apply this.
- You discuss here, “Metarhizium pingshaense could be used as an alternative to replace chemical insecticides;”. This is not correct. The goal of IVM isn’t to replace what works but complement existing methods.
- You mention here, “….however, their combination may offer strong synergy..” I don’t think it would be feasible to combine them since you would have to find a way to infect mosquitoes first for days before exposing them to insecticides. Please discuss more.
- You mention here, “…..improving Metarhizium virulence against insecticide-resistant mosquitoes.” I thought Metarhizium improves insecticide lethality. Clarify!
- In the statement, “However, it is necessary to better understand the synergy observe in ..” there is a grammatical error here (observed nor observe)

**General comments:**

- If *Metarhizium *occurs naturally in mosquito species, then why isn’t this synergistic effect observed in wild resistant mosquito populations? The discussion needs to come out clearly.
- Parts of the discussion need to be re-written. Some of the paragraphs are confusing to read and there are no connecting words or sentences. It is hard to follow. Furthermore, the discussion is a bit shallow and could use more explanations.

Is the work clearly and accurately presented and does it cite the current literature?

Partly

If applicable, is the statistical analysis and its interpretation appropriate?

Partly

Are all the source data underlying the results available to ensure full reproducibility?

Yes

Is the study design appropriate and is the work technically sound?

Partly

Are the conclusions drawn adequately supported by the results?

No

Are sufficient details of methods and analysis provided to allow replication by others?

Partly

Reviewer Expertise:

Vector Biology, Molecular Genetics and Field trials and operational research.

I confirm that I have read this submission and believe that I have an appropriate level of expertise to confirm that it is of an acceptable scientific standard, however I have significant reservations, as outlined above.

# Reviewer report: Reviewer response for version 1

**DOI:** 10.21956/wellcomeopenres.23487.r91453

**Published:** 2024-08-28

**Authors:** Shüné Oliver

**Affiliations:** 1 Division of the National Health Laboratory Service, National Institute for Communicable Diseases, Johannesburg, South Africa; 2 University of the Witwatersrand Johannesburg, Johannesburg, Gauteng, South Africa

The authors provide evidence of the synergism of local fungi and deltamethrin with the aim of dealing the problem of insecticide resistance. Although I do not really have a problem with the science, there are quite a few things that must be addressed before indexing.

**I have made various comments on the PDF, but my general comments are as follows.**

**Please view the additional review comments in the file found** here .

**Plain summary:**

 I have highlighted this on the text, but this is still too “jargoney” for a plain summary. This is common, as although the summary is simple enough for a scientist, it is still a little too technical for a non-specialist audience.

**Introduction:**

 Again, highlighted on the MS, but there needs to be tightening up of phrasing and some language and grammar will improve the MS. Please be careful of the very long sentences, as it makes it difficult to follow. There are a few places that language needs to be attended to, but in general, the language is not too bad and is not unreadable, there are just a few points that need to be fixed.

**Methods:**

 A few places highlighted on the MS, but more detail is needed to allow replication.

**Results:**

 One highlighted legend needs to be clarified.

**Discussion:**

 I am generally happy with the discussion, but there are two major issues that need to be attended to. Firstly, there needs to be more thought on how the combination of the fungus and insecticide are to be deployed. The fungus impregnated ITN has a range of issues. How will the net maintain the fungus when washed? Also, as I highlighted in the introduction (and this is the second part of the issue), Metarhizium is not innocuous and can infect people. This is going to be a problem in Africa where immunocompromise is an issue. Will nets be the best way to do this? Also, how does this approach give an initial inoculum of fungus (which the authors highlight is important, as this order is crucial)? Basically, without giving a suggestion of how this knowledge is to be applied, this will be replicating data already known rather than giving new data. I would also suggest a better discussion of the importance of the use of a native fungus rather than a generalised fungus would strengthen the discussion.

Is the work clearly and accurately presented and does it cite the current literature?

Yes

If applicable, is the statistical analysis and its interpretation appropriate?

Yes

Are all the source data underlying the results available to ensure full reproducibility?

Yes

Is the study design appropriate and is the work technically sound?

Yes

Are the conclusions drawn adequately supported by the results?

Yes

Are sufficient details of methods and analysis provided to allow replication by others?

Partly

Reviewer Expertise:

Malaria vector biology, Insecticide resistance.

I confirm that I have read this submission and believe that I have an appropriate level of expertise to confirm that it is of an acceptable scientific standard, however I have significant reservations, as outlined above.

# Response

**Published:** 2024-09-03

**Authors:** Etienne Bilgo

**Affiliations:** Public Heath and Biomedical, Institut de Recherche en Sciences de la Santé, Bobo Dioulasso, Houet, Burkina Faso

Dear Editor,

 Thank you very much for the reviews. We have addressed all the comments and suggestions from the reviewers and are pleased to submit a revised version of the manuscript for your consideration. 

 Best regards,

 Etienne
